# The Plasminogen Activation System Modulates Differently Adipogenesis and Myogenesis of Embryonic Stem Cells

**DOI:** 10.1371/journal.pone.0049065

**Published:** 2012-11-08

**Authors:** Ola Hadadeh, Emilie Barruet, Franck Peiretti, Monique Verdier, Denis Bernot, Yasmine Hadjal, Claire El Yazidi, Andrée Robaglia-Schlupp, Andre Maues De Paula, Didier Nègre, Michelina Iacovino, Michael Kyba, Marie-Christine Alessi, Bernard Binétruy

**Affiliations:** 1 Inserm U626, Faculté de Médecine, Marseille, France; 2 Aix-Marseille Université, Faculté de Médecine, Marseille, France; 3 Inserm U910, Faculté de Médecine, Marseille, France; 4 Laboratoire de Biologie Cellulaire, CHU (Centre Hospitalier Universitaire) La Timone AP-HM (Assistance Publique – Hôpitaux de Marseille), Marseille, France; 5 Service d’Anatomie Pathologique et Neuropathologie, CHU (Centre Hospitalier Universitaire) La Timone AP-HM (Assistance Publique – Hôpitaux de Marseille), Marseille, France; 6 INSERM, U758, Ecole Normale Supérieure de Lyon, Lyon, France; 7 Université de Lyon, UCB-Lyon1, Ecole Normale Supérieure de Lyon, Lyon, France; 8 Department of Pediatrics and Lillehei Heart Institute, University of Minnesota, Minneapolis, Minnesota, United States of America; William Harvey Research Institute, Barts and The London School of Medicine and Dentistry, Queen Mary University of London, United Kingdom

## Abstract

Regulation of the extracellular matrix (ECM) plays an important functional role either in physiological or pathological conditions. The plasminogen activation (PA) system, comprising the uPA and tPA proteases and their inhibitor PAI-1, is one of the main suppliers of extracellular proteolytic activity contributing to tissue remodeling. Although its function in development is well documented, its precise role in mouse embryonic stem cell (ESC) differentiation in vitro is unknown. We found that the PA system components are expressed at very low levels in undifferentiated ESCs and that upon differentiation uPA activity is detected mainly transiently, whereas tPA activity and PAI-1 protein are maximum in well differentiated cells. Adipocyte formation by ESCs is inhibited by amiloride treatment, a specific uPA inhibitor. Likewise, ESCs expressing ectopic PAI-1 under the control of an inducible expression system display reduced adipogenic capacities after induction of the gene. Furthermore, the adipogenic differentiation capacities of PAI-1^−/−^ induced pluripotent stem cells (iPSCs) are augmented as compared to wt iPSCs. Our results demonstrate that the control of ESC adipogenesis by the PA system correspond to different successive steps from undifferentiated to well differentiated ESCs. Similarly, skeletal myogenesis is decreased by uPA inhibition or PAI-1 overexpression during the terminal step of differentiation. However, interfering with uPA during days 0 to 3 of the differentiation process augments ESC myotube formation. Neither neurogenesis, cardiomyogenesis, endothelial cell nor smooth muscle formation are affected by amiloride or PAI-1 induction. Our results show that the PA system is capable to specifically modulate adipogenesis and skeletal myogenesis of ESCs by successive different molecular mechanisms.

## Introduction

Adipose, vascular and muscle tissue plasticity occurs in a number of normal and physiopathological processes including embryonic development, adult muscle aging, muscular dystrophies, obesity and diabetes. There is ample evidence both *in vivo* and *in vitro* that the extracellular matrix (ECM) surrounding the tissues plays an important functional biological role either in physiological or pathological conditions. Likewise its function in development is well documented, suggesting that ECM could be involved in the regulation of the commitment and differentiation of stem cells. The concept of niche in which the stem cell fate is dependent on interactions with its surrounding environment represents a paradigm of these regulations [1], [2].

The PA system is one of the main suppliers of extracellular proteolytic activity, such as the fibrinolysis, thus contributing to ECM degradation and tissue remodelling. It is exerted by two distinct plasminogen activators, tPA (tissue-type plasminogen activator) and uPA (urokinase-type plasminogen activator) [3], [4]. The plasminogen activator inhibitor-1 (PAI-1) directly inhibits the proteolytic activity of uPA and tPA [3]. This system is implicated in numerous fibrinolytic and non-fibrinolytic processes leading to either ECM degradation, activation of latent growth factors, or integrin-mediated cell adhesion on vitronectin (VN) [5], [6]. All these processes are inhibited by PAI-1 [7], [8]. In vitro, PAI-1 regulates adipocyte differentiation [9] and, by controlling endothelial cell migration and prolifereation, is a potent regulator of angiogenesis [10], [11], [12]. Finally, PAI-1 interferes with myogenesis: myogenic cells express the proteins of the plasminogen activation system [13], [14] and uPA is able to stimulate proliferation, migration and fusion of satellite cells, whereas uPA inhibition abrogated myogenesis [15]. In addition, genetic approaches demonstrated that uPA, but not tPA, activity is required for efficient skeletal muscle regeneration in vivo [16] and that this process is accelerated in PAI-1-deficient mice [17].

However the precise role of the PA system and more generally the role of ECM during the development and the commitment of pluripotent cells in *in vitro* experimental models such as the differentiation of Embryonic Stem Cells (ESCs) are still poorly known. ESCs are pluripotent and can be induced to differentiate into a variety of cell types [18]. Transplantation of ESCs has been proposed as a future therapy for various human diseases. However only variable fractions of cells reaches the desired differentiated phenotype, therefore their therapeutic use necessitates a better understanding of the molecular mechanisms that control their commitment. Self-renewal of ESCs is dependent on a complex interplay between specific stimuli (for example, the growth factor LIF for mouse ESCs) specific epigenetic processes, miRNAs and transcription factors involved in the development of the embryo. Appropriate culture conditions result in their commitment into mature differentiated cell types (see for reviews [18], [19], ). In order to approach the potential regulatory role of extracellular matrix, we studied the plasminogen activation (PA) system during ESC differentiation.

We found that the PA system components are expressed at very low levels in undifferentiated ESCs and that upon differentiation uPA activity is detected mainly transiently, whereas tPA activity and PAI-1 protein are maximum in well differentiated cells. We then characterized the role of the PA system in ESC differentiation by different approaches and found that interfering with uPA activity leads, on the one hand, to inhibition of adipogenesis and, on the other hand, to positive and negative regulations of skeletal myogenesis by successive different molecular mechanisms.

## Materials and Methods

### Differentiation of ESCs

CGR8 mouse ESCs [21] were grown on gelatin-coated plates and A2lox.cre ESCs [22] were grown on MEF feeders treated with 10 µg/ml mitomycin C for 3 hours. 1×10^3^ ESCs were aggregated without LIF to form embryoid bodies (EB) at day 0, cultivated in suspension until day 7, treated or not with 10^−7 ^M All-Trans Retinoic Acid (Sigma, France) daily between days 3 to 5, plated at day 7 on gelatin-coated 6-well plate and treated with 85 nM insulin (Sigma, France), 2 nM 3,3′,5-tri-iodothyronine (Sigma, France) to induce terminal differentiation. Fresh medium containing the inducers was applied every 2 days. EBs were analyzed for cardiomyocytes, endothelial cells or neurons at day 12, and adipocytes or myotubes at day 26, as described previously [21], [23], [24]. 100 µmol/L of the uPA inhibitor Amiloride (Sigma, France) or 0.5 µg/ml of doxycycline (Sigma, France) were added to the culture media at different period of time as indicated.

### Plasmid and Generation of ESC Lines

After addition of two HindIII sites, the human PAI-1 cDNA isolated from the PIGE20-hPAI-1-stable [25] was inserted into the p2Lox [22], generating the p2Lox-PAI-1 plasmid. The A2lox.cre ESC line was electroporated with 2 µg of p2Lox-hPAI-1 (Amaxa Biosystems, A24 program for ESC), according to the manufacturer’s protocol, and selected with 500 µg/ml G418 (PAA, France). Transfection was realized onto mitomycin C-treated stable neomycin resistant MEF feeder cells obtained by transduction with the CHC-vector, a MLV retrovirus-vector coding the Neo cDNA using an MOI of 6 UI/cell, followed by Neomycin selection (100 µg/ml) as described previously [26].

### Generation of iPSCs

1×10^5^ wt or PAI-1^−/−^ MEFs [27] cultivated in DMEM supplemented with 10% fetal bovine serum were infected, at 30xMOI, with the KOSM polycistronic lentivirus vector encoding the murine transcriptor factors Klf4, Oct4, Sox2 and Myc [28]. The day after, medium was changed for the ESC medium. Colonies presenting an ESC phenotype were visible after 2 weeks and picked at day19.

### RNA analysis

Total RNA was prepared using Trizol reagent (Invitrogen, France). Real time Reverse Transcriptase-PCR was performed with the ABI Prism7300 (Applied BioSystems, France) and Mesa Green MasterMix (Eurogentec, France). The relative amounts of the different mRNAs were quantified by using the comparative CT method (2-(ΔΔCT)). 36B4 was used as housekeeper transcripts and gene expressions were normalized using 36B4 RNA levels. Primer sequences are given in **Data S12**.

### Western Blot Analysis

ESCs were lysed as described [21] at days 0 (for Oct4 analysis) or 24 (for MyH1) of the differentiation protocol. Samples at day 0 (20 µg) were separated by SDS-polyacrylamide gel electrophoresis on a 10% gel (Invitrogen, France), transferred onto polyvinylidene difluoride membranes (Millipore, France) and incubated with antibodies against Oct-4 (1∶1000, Abcam, France) or ERK (1∶2000, Santa Cruz, California, USA). Samples at day 24 (20 µg) were separated by SDS-polyacrylamide gel electrophoresis on a 3–8% gel (Invitrogen, France), transferred onto polyvinylidene difluoride membranes (Millipore, France) and incubated with antibodies against Myosin (MF20) (1∶500, DSHB, Iowa City, USA) or ERK.

### Immunocytochemistry, Immunofluorescence and Oil Red O Staining

For **PAI-1 immunocytochemistry** studies, cells were washed at day 24, fixed in 4% paraformaldehyde for 15 min at room temperature (RT), permeabilized in PBS-0.2% Triton X-100 for 15 min at RT, blocked by serum for 30 min, and then incubated with 23.4 µg/ml rabbit anti-mouse PAI-1 (H34G6D10, provided by Dr P. Declerck) for 1 h. After rinsing twice with PBS, anti-rabbit IgG secondary antibody (Vector, UK) was added, incubated for 40 min, followed by two washes in PBS, incubated with vectastain ABC (Vector, UK) for 30 min, washed with PBS, and stained with DAB (Dako, France). Slides were counterstained with hematoxylin (1∶2 dilution in distilled water).

For **MyH1 immunofluorescence** studies, fixed cells at day 24 were permeabilized in PBS-0.2% Triton X-100 for 15 min at RT and incubated with anti-Myosin (MF20) (DSHB, Iowa City, USA) in PBS-10% FBS for 2 hours. After three washes in PBS, cells were incubated with anti-mouse ALEXA488 (Invitrogen, France) for 1 hour at RT.

For **ECM characterization**, cell cultures were first acellularized by using a cultured cells Acellularization kit (Sigma, France). Briefly, EBs at days 12 and 24 were incubated for 10 min with the Acellularization buffer and gently shaked at RT. After carefully aspirating the Acellularization buffer, cultures were carefully rinsed twice with PBS. The ECM was then incubated with anti-Collagen I (1∶100, Abcam, France) in PBS-1% BSA for 2 hours at RT. After three washes in PBS, ECM was incubated with anti-rabbit ALEXA468 (Invitrogen, France) for 1 hour at RT.

For **iPSC characterization**, fixed cells at day 0 were permeabilized in PBS-0.2% Triton X-100 for 15 min at RT and incubated with either anti-SSEA1 (MC480) (DSHB, Iowa City, USA), anti-Sox2 (AB5603) (Millipore, France), anti-Oct4 (ab19857) (Abcam, France), or anti- Nanog (SC30328) (Santa Cruz Biotechnology, California, USA) in PBS-10% FBS for 2 hours. After three washes in PBS, cells were incubated with either anti-mouse ALEXA488 (Invitrogen, France), anti-rabbit ALEXA468 (Invitrogen, France), or anti-goat ALEXA568 (Invitrogen, France) for 1 hour at RT.

For **Oil Red O Staining**, fixed cells at day 24 were immersed 30 min at RT in a solution of Oil Red O [29], and then washed 5 times with deionised water.

### Flow Cytometry

Cells were trypsinized, washed with 10%FBS/DMEM, fixed in 0,5% paraformaldehyde for 15 min at room temperature (RT) and permeabilized with 0.1% Triton/PBS buffer for 10 min at RT. Cells were then stained with an α-Myogenin (FD5) antibody at 1∶10 dilution in 5%FBS/PBS (DSHB, Iowa City, USA) for 1 hour under agitation, washed with 5%FBS/PBS, and incubated with an anti-mouse IgG secondary antibody conjugated to PE (1∶100 dilution in 5%FBS/PBS) (Beckman Coulter, France) for 30 min under agitation. After additional washes in PBS, cells were resuspended in 500 µl PBS and analyzed on a FC500 Flow cytometer using CXP software (Beckman Coulter, France). For each sample, at least 5000 cells were analyzed. Cursor for positive fluorescent cell determination was placed, such as 75% of events were outside (first decade) the cursor region when an isotype IgG antibody was used. Results are given as percentage corresponding to positive fluorescent cells included in the region defined by the cursor and after subtracting the irrelevant’s percentage.

### ELISA Assays

**Murine uPA and tPA antigens** were quantified using respectively the MUPAKT-TOT and MTPAKT-TOT immunoassay kits from Gentaur, France, according to supplier’s recommendations. A standard calibration curve was prepared using dilutions of purified uPA from 0.025 to 2 ng/ml and of purified tPA from 0 to 50 ng/ml.

**Murine uPA and tPA activities** were quantified using respectively the MUPAKT and MTPAKT immunoassay kits from Gentaur, France, according to supplier’s recommendations. A standard calibration curve was prepared using dilutions of purified uPA from 0.025 to 2 ng/ml and of purified tPA from 0 to 50 ng/ml.

**Human and mouse PAI-1 antigens** were quantified by immunoassays, wells of microtiter plates were coated overnight at 4°C with 2 µg/ml human PAI-1 antibody (15H12, provided by Dr P. Declerck), or 48 h at 4°C with 2 µg/ml rabbit anti-mouse PAI-1 (H34G6D10 provided by Dr P. Declerck), washed with PBS 0,004% Tween 80, and blocked for 2 h with a solution of 1% BSA in PBS. Cell lysates, diluted five times, or undiluted conditioned media, prepared from 24 hours cell supernatants cultivated without serum, were added to each well and incubated overnight at 4°C. After four washes with PBS 0,004% Tween 80, biotinylated anti-mPAI-1 antibody Ra-mPAI-1(1∶800) was added for 1 h at RT, and for another hour with vectastain ABC (Vector, UK), or anti-human antibody 12A4A6 (1∶800) was added for 2 h at RT. After four washes with PBS 0,004% Tween 80, ultra-TMB solution (Thermo scientific, France) was added. The reaction was stopped by adding H2SO4 (2M final concentration), and the optical density was read at 450 nm. A standard calibration curve was prepared using dilutions of purified PAI-1 from 0 to 10 ng/ml.

**Human PAI-1 activity** was quantified by using the Zymutest PAI-1 Activity immunoassay kit (HYPHEN Biomed, France); according to supplier’s recommendations. A standard calibration curve was prepared using dilutions of purified PAI-1 from 0 to 20 ng/ml.

### Teratoma Formation and Histological Analyses

CGR8 ESCs, and wt and PAI-1^−/−^ iPSCs were trypsinized and washed, and 5×10^6^ cells resuspended in 200 µl of PBS were injected subcutaneously into both sides of the back of 6-week-old athymic nude female mice (Harlan, France). At 5 weeks following injection, tumours were surgically excised, fixed in formalin, paraffin-embedded, and 5 *µ*m thick sections were analyzed and photographed after hematoxylin–eosin staining.

### Statistical Analyses

All experiments were performed at least three times. All data are expressed as the mean ±SEM. Treatments were compared with their respective controls and significant differences among the groups were determined using unpaired Student’s t-test. A value of p<0.05 was taken as an indication of statistical significance.

## Results

### Gene Expression of uPA, tPA,uPAR and PAI-1 before and after ESC Differentiation

Mouse ESCs are differentiated by removing the LIF from the medium and forming aggregates in suspension, called embryoid bodies (EBs), for one week. After this crucial commitment phase, cells are allowed to terminally differentiate by replating EBs on Petri dishes. This experimental protocol leads to the spontaneous formation, into the same culture dishes and in function of time, of several well-differentiated cells from a fraction of the starting ESC population. Neuron and adipocyte formation necessitates a critical early three days treatment of EBs between days 3 to 5 of the protocol with retinoic acid (hereafter referred as RA or adipogenic conditions); conversely, not treated RA EBs will form spontaneously endothelial cells, cardiomyocytes, and smooth and skeletal myogenic cells (hereafter referred as NoRA or myogenic conditions). Routinely, at day 12 of differentiation, we followed neurogenesis by the expression of the neuronal marker MAP2; endothelial cell differentiation by the expression of Flk-1, PECAM and von Villebrand Factor (vWF) genes; smooth muscle cells by the alpha-smooth muscle actin (α-SMA) marker; and cardiomyogenesis by the TroponinT and GATA4 markers. At day 24 of differentiation, we followed adipogenesis by the expression of aP2, PPARgamma and adiponectin genes and skeletal myogenesis by the Myogenin and Myosin heavy Chain-1 (MyH1) markers [26], [30], [31].

**Figure 1:**
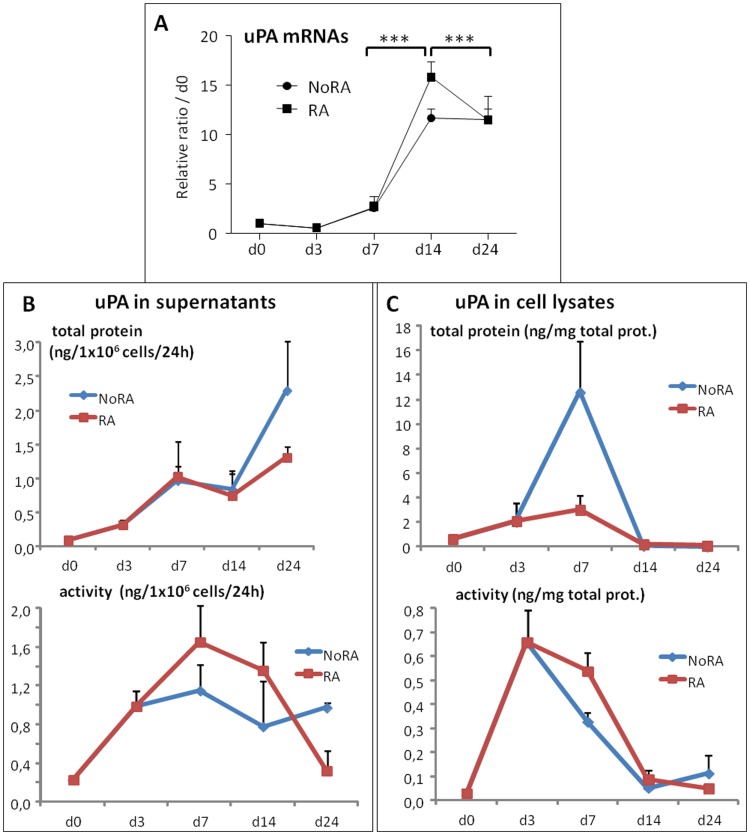
endogenous uPA mRNA, protein expression and enzymatic activities during ESC differentiation. Retinoic acid-treated (RA) or not (NoRA) EBs from wild type CGR8 ESCs were induced to differentiate and analyzed at various time, as indicated, between days 0 (**d0**) to 24 (**d24**). (**A**) mRNAs were extracted and analyzed by real time RT-PCR for the expression of uPA. Results are expressed in arbitrary units, with the values of wild type CGR8 at day 0 taken as 1, and are the means ± S.E.M. of at least 3 independent experiments. Significance is given as: *P<0.05, **P<0.01 and ***P<0.001. (**B and C**) uPA antigen (upper panels) or specific uPA enzymatic activity (lower panels) were quantified by ELISA technique in either total cell lysate protein extracts (**B**) or in 24 hr. conditioned medium of cells cultivated without serum (**C**). Mean values of at least three independent experiments are given.

**Figure 2:**
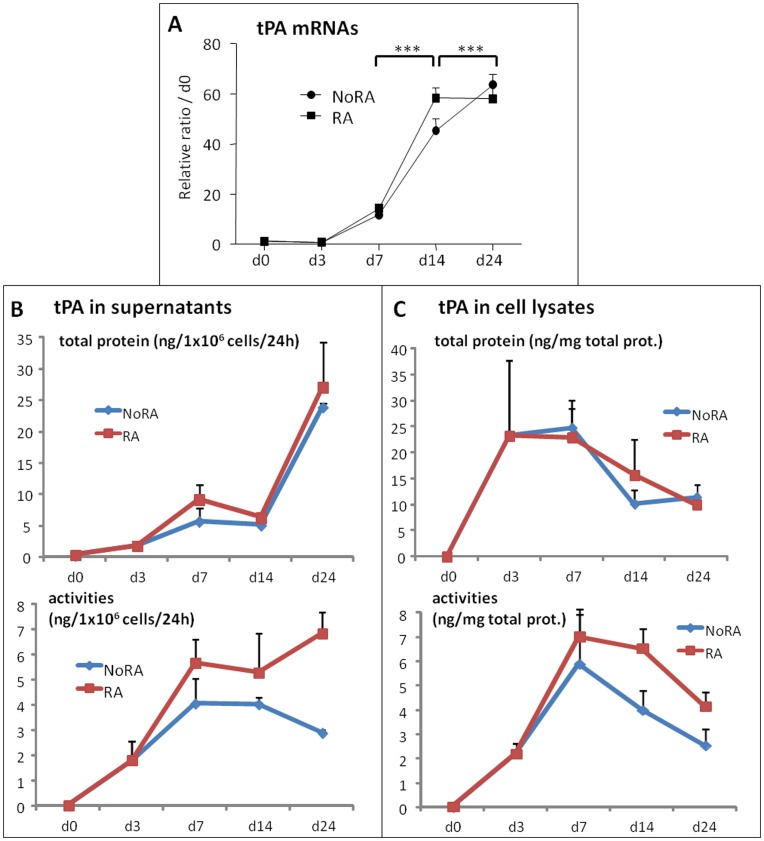
endogenous tPA mRNA, protein expression and enzymatic activities during ESC differentiation. Retinoic acid-treated (RA) or not (NoRA) EBs from wild type CGR8 ESCs were induced to differentiate and analyzed at various time, as indicated, between days 0 (**d0**) to 24 (**d24**). (**A**) mRNAs were extracted and analyzed by real time RT-PCR for the expression of tPA. Results are expressed in arbitrary units, with the values of wild type CGR8 at day 0 taken as 1, and are the means ± S.E.M. of at least 3 independent experiments. Significance is given as: *P<0.05, **P<0.01 and ***P<0.001. (**B and C**) tPA antigen (upper panels) or specific tPA enzymatic activity (lower panels) were quantified by ELISA technique in either total cell lysate protein extracts (**B**) or in 24 hr. conditioned medium of cells cultivated without serum (**C**). Mean values of at least three independent experiments are given.

**Figure 3:**
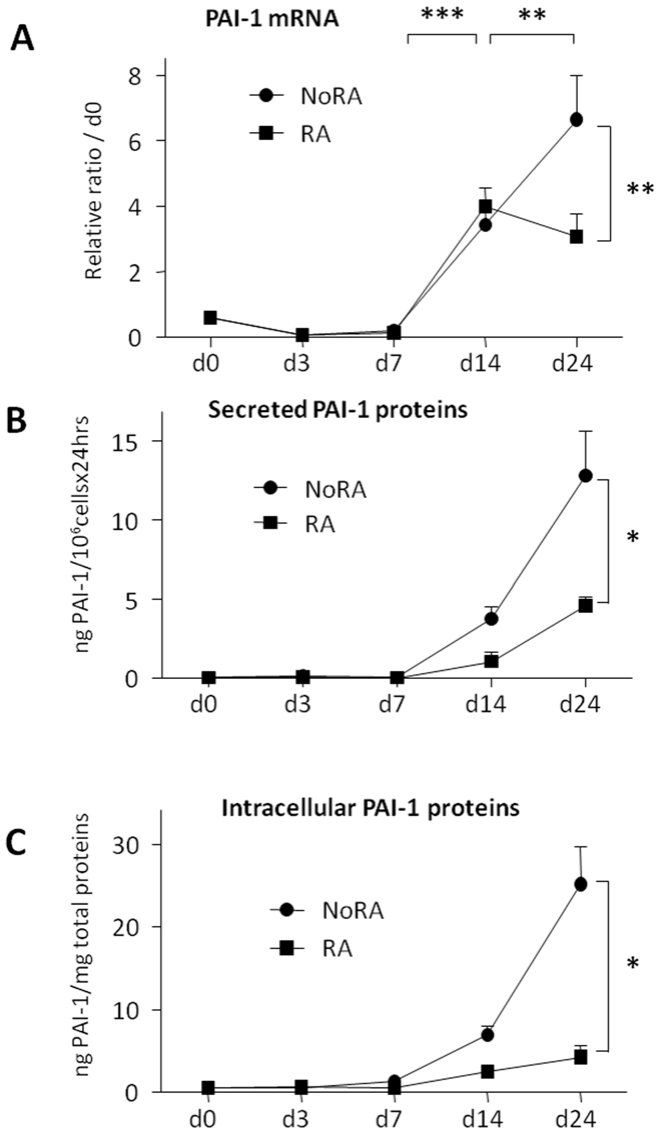
endogenous PAI-1 mRNA and protein expression during ESC differentiation. Retinoic acid-treated (RA) or not (NoRA) EBs from wild type CGR8 ES cells were induced to differentiate and analyzed at various time, as indicated, between days 0 (**d0**) to 24 (**d24**). (**A**) mRNAs were extracted and analyzed by real time RT-PCR for the expression of PAI-1. Results are expressed in arbitrary units, with the values of wild type CGR8 at day 0 taken as 1, and are the means ± S.E.M. of at least 3 independent experiments. Significance is given as: *P<0.05, **P<0.01 and ***P<0.001. (**B** **and** **C**) PAI-1 protein expression in total cell lysates (**B**) or conditioned medium (**C**) was quantified by ELISA technique at various time, as indicated, between days 0 (**d0**) to 24 (**d24**). Values are given in ng of PAI-1 amounts and expressed as means of at least three independent experiments ± S.E.M. Significance between RA and NoRA treatments is given.

mRNAs, proteins and enzymatic activities of uPA and tPA have been analyzed by quantitative RT-PCR and ELISA techniques at various times during the differentiation of CGR8 mouse ESCs. uPA and tPA expressions and activities are almost undetectable in undifferentiated ES cells (**Fig. 1** and **2**). Upon differentiation, with or without RA treatment, uPA mRNA expression increases gradually, reaching a maximum at day 14 (**Fig. 1A**). A similar expression profile is found for uPA proteins in cell supernatants, except for the NoRA myogenic condition where it continues to augment till day 24 (**Fig. 1B**). Interestingly, uPA activities in supernatants, although following an identical profile until day 14, are then lower. This latter enzymatic inhibition could be due to the presence of high amounts of PAI-1 proteins during this period (**Fig. 3B**). Intracellular uPA protein and activity are detected only transiently during the differentiation process, at days 3 and 7, being almost undetectable by day 14 (**Fig. 1C**).

Upon differentiation, with or without RA treatment, tPA protein expression in supernatants and tPA mRNAs increase gradually, reaching a maximum at day 24 (**Fig. 2A and B**). tPA activities in supernatants, although globally following an identical profile to uPA activities do not decrease significantly after day 14, staying at high levels until day 24. Intracellular tPA protein and activity are detected from days 3 to 24, reaching a maximum at day 3 and then slightly decreasing (**Fig. 2C**).

Expression of PAI-1 (mRNA and protein) is almost undetectable in undifferentiated cells and till day 7 and correlates mainly with terminal adipocyte and skeletal myogenic differentiations (**Fig. 3**). Interestingly, at day 24 of the differentiation, PAI-1 expression is significantly higher in cultures without RA treatment than in RA-treated embryoid bodies, suggesting that PAI-1 expression could be higher in skeletal myotubes than in adipocytes. Since these cultures are not pure differentiated cell populations, one could hypothesize that PAI-1 would not be expressed in adipocytes. Therefore, we analyzed the expression of PAI-1 by immunocytochemistry at day 24 in well differentiated cells. In all tested conditions, we were able to detect PAI-1 protein in both adipocytes and myotubes (**Data S1**).

Variations of expression of the receptor of uPA, uPAR, are very limited during the differentiation process (data not shown).

Altogether these results show that uPA activity is mainly present during the early d0–d14 period, whereas tPA activity is still strongly detected in well differentiated cells; PAI-1 protein is present only in the latter steps of differentiation, from d14 to d24.

### uPA Inhibition by Amiloride Decreases ESC Adipogenesis

**Figure 4:**
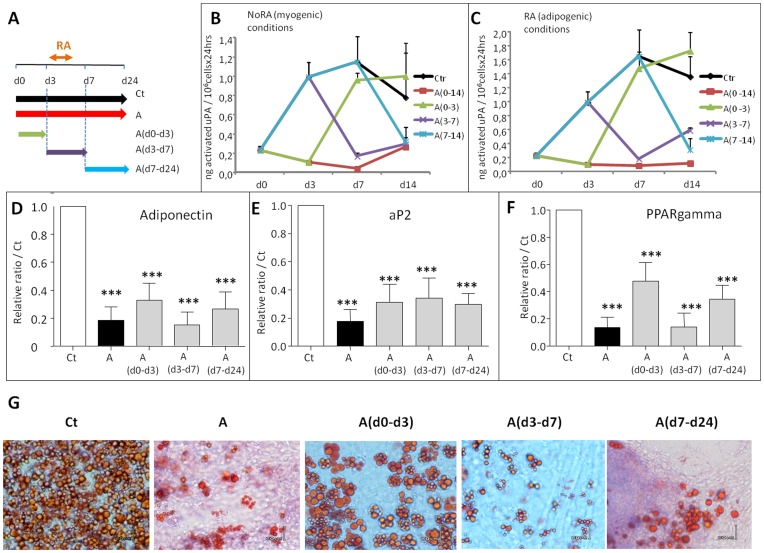
inhibition of uPA by amiloride treatments interfere with ESC adipogenesis. EBs from wild type CGR8 ES cells were induced to differentiate and treated or not by 100 µM amiloride for different period of time: from days 0 to 24 **[A]**, from days 0 to 3 **[A(d0–d3)]**, from days 3 to 7 **[A(d3–d7)]**, from days 7 to 14 **[A(d7–d14)]** or 24 **[A(d7–d24)].** (**A**) Scheme of the different amiloride treatments. (**B and C**) ELISA assay quantification of mouse activated uPA into the 24hrs conditioned medium of 10^6^ cells RA-treated (**C**, adipogenic conditions) or not (**B**, skeletal myogenic conditions) and treated or not by amiloride. (**D to G**) Effects of amiloride treatments on ESC adipogenesis. mRNAs from retinoic acid-treated (adipogenic conditions) EBs were extracted and analyzed by real time RT-PCR for the expression of the adipogenic markers adiponectin (**D**), aP2 (**E**) and PPARgamma (**F**). Results are expressed in arbitrary units, with the values of untreated CGR8 at day 24 taken as 1, and are the means ± S.E.M. of at least 3 independent experiments. Significance to Ct values is given as: *P<0.05, **P<0.01 and ***P<0.001. (**G**) Adipocyte formation of CGR8 treated or not by amiloride, as indicated, was visualized by Oil RedO staining, representative fields are shown.

These striking regulations of endogenous components of the PA system during ESC differentiation suggested important biologic roles for these molecules. In order to analyze their regulatory potential, we treated ESCs with amiloride, a potent specific chemical uPA inhibitor which does not affect the tPA protease [32]. Because the first week of the differentiation process is critical for the ESC commitment [26], 100 µM of the inhibitor were applied on CGR8 ESCs in four different conditions: i) all the time of differentiation, from days 0 to 24 **[A]**, ii) only from days 0 to 3 **[A(d0–d3)]**, iii) from days 3 to 7 **[A(d3–d7)]**, iv) from days 7 to 24 **[A(d7–d24)]** (**Fig. 4A**). The inhibitory effect of amiloride on uPA enzymatic activity was measured between days 0 to 14 of the differentiation process. Whereas uPA activity is very low in control undifferentiated cells, this activity augments as early as day 3, reaching a maximum, at day 7 into the 24 hrs conditioned medium of RA-treated (adipogenic conditions) or not (skeletal myogenic conditions) cells, respectively (**Fig. 4B and C**, black curves). These activities are completely inhibited by the amiloride treatment all the time of differentiation, (red curves), inhibition at day 14 is also observed when amiloride is added at day 7 (blue curves). Interestingly, when amiloride is added only between days 0 to 3, uPA activity is inhibited at day 3 but is fully recovered at days 7 and 14 (green curves). Likewise, when amiloride is added between days 3 to 7, uPA activities decrease during this period and are partially recovered by day 14 (violet curves). Importantly, these results demonstrate that the uPA inhibition by amiloride is a reversible process. Although close to the detection limit of the assay, similar results were obtained by analyzing uPA activity in cellular lysates (**Data S2**).

We then analyzed the effects of the inhibitor on ESC differentiation. All amiloride treatments of CGR8 cells significantly decrease by more than 60% the expression of adipogenic markers measured at day 24: adiponectin (**Fig. 4D**), aP2 (**Fig. 4E**) and PPARgamma (**Fig. 4F**). These inhibitions of adipocyte formation resulted in less lipids accumulated in cell cultures, as detected by Oil RedO staining (**Fig. 4G**). As a control, we found no effect on cells treated with di-methyl-amiloride, an amiloride-derived compound which no longer inhibits uPA (data not shown). Since uPA activities are detected at least until day 14, these results suggest that uPA plays important successive roles during the whole process of ESC adipogenesis.

In order to check whether the amiloride treatment could affect the extracellular matrix (ECM) secreted by ESCs during the differentiation process, we characterized the collagen 1 ECM composition by immunofluorescence studies after acellularization of the cultures at days 12 and 24. Collagen 1 is detected in low amounts at day 12 of differentiation in control cells, treated (**Data S3**) or not (**Data S4**) by RA and its expression is slightly augmented by day 24. Interestingly, the amiloride treatment A(d0–d24) strongly increases collagen 1 deposition at day 24 in both RA and NoRA treated cells and this increase is visible as soon as day 12 in the NoRA condition. This effect is reproduced (although to a lesser extend in the NoRA condition) by the A(d7–d24) treatment. These results suggest that uPA inhibition by amiloride leads to ECM accumulation in differentiating ESCs. We can hypothesize that this effect is mediated by interference with the uPA-plasmin-matrix metalloproteases cascade which induces ECM degradation [33]. The early amiloride treatments A(d0–d3) and A(d3–d7) have no significant effect on ECM deposition, examined at day 24; whereas they augment collagen 1 deposition at day 12. It is important to note that all the amiloride treatments are equally inhibitor regarding adipocyte differentiation; therefore it is possible that the collagen 1 regulation by amiloride contributes to its effect on differentiation but is neither sufficient nor necessary.

### Construction of an ESC Line Expressing Ectopically PAI-1 under an Inducible Control

In order to by-pass a potential effect of amiloride unrelated to uPA inhibition, we undertook a genetic approach. We used a sophisticated Tet-On inducible system adapted to mouse ESCs described in Iacovino et al. [22]. Upon cre-lox recombination, the mouse ESC line A2lox.cre, allows the reproducible targeting of a locus on the X-chromosome, in the region upstream of the Hprt gene, giving robust doxycycline-inducible expression of genes of interest in both mESCs and differentiated cells.

**Figure 5:**
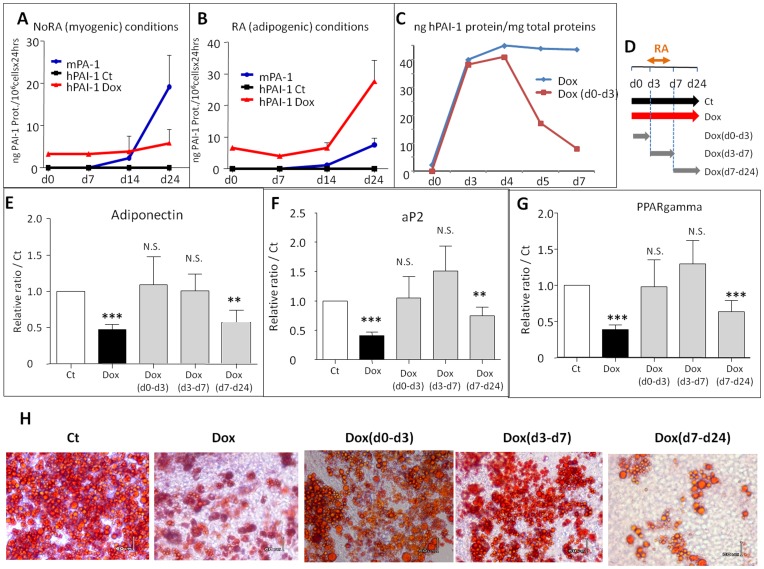
Doxycycline-induced human PAI-1 expression inhibits ESC adipogenesis of the A2lox.cre mESC clone3. (**A and B**) Retinoic acid-treated (**RA**, panel **B**) or not (**NoRA,** panel **A**) EBs from A2lox.cre mESC clone3 cells treated (**Dox**, red lines) or not (**Ct**, black lines) by 0.5 µg/ml of doxycycline were induced to differentiate and analyzed at various time, as indicated, between days 0 (**d0**) to 24 (**d24**). Endogenous mouse (mPAI-1, blue lines) and ectopic human (hPAI-1) PAI-1 protein expressions in conditioned medium were quantified by ELISA technique. Values are given in ng of PAI-1 amounts and expressed as means of at least three independent experiments ± S.E.M. (**C**) Evaluation of the turnover of Dox-induced intracellular human PAI-1 by ELISA after doxycycline removal. ESCs were treated with doxycycline either all the time (blue line) or between days 0 to 3 (red line) and hPAI-1 protein was analyzed until day 7. Values are given in ng of human PAI-1 amounts/mg of total proteins. Retinoic acid-treated EBs from A2lox.cre mESC clone3 cells were induced to differentiate to adipocyte and treated or not by doxycycline for different period of time: from days 0 to 24 **[Dox]**, from days 0 to 3 **[Dox(d0–d3)]**, from days 3 to 7 **[Dox(d3–d7)]**, from days 7 to 24 **[Dox(d7–d24)].** (**D**) Scheme of the different doxycycline treatments. (**E to G**) mRNAs were extracted and analyzed by real time RT-PCR for the expression of the adipogenic markers adiponectin (**E**), aP2 (**F**) and PPARgamma (**G**). Results are expressed in arbitrary units, with the values of untreated A2lox.cre mESC clone3 cells at day 24 taken as 1, and are the means ± S.E.M. of at least 3 independent experiments. Significance to Ct values is given as: N.S. non significant, *P<0.05, **P<0.01 and ***P<0.001. (**H**) Adipocyte formation of A2lox.cre mESC clone3 cells treated or not, as indicated, by doxycycline was visualized by Oil RedO staining, representative fields are shown.

We first analyzed the time-course expression of the endogenous PA components in A2lox.cre ESCs before and after differentiation. Similar results to what observed in CGR8 cells were obtained (**Data S5**). The human PAI-1 cDNA was cloned into the targeting vector and we isolated A2lox.cre ESC clones expressing human PAI-1 after doxycycline induction. We further characterized the clone 3 that expresses high levels of hPAI-1. As expected, clone 3 do not express human PAI-1 protein without doxycycline induction, either intracellularly (not shown) or within its conditioned medium, and before or after differentiation with or without RA treatment (**Fig. 5** **A and B**, black curves). By contrast, significant human PAI-1 protein levels are constantly produced after induction during the 24 days of the differentiation protocol (**Fig. 5A and B**, red curves). Importantly, in undifferentiated ESCs and during the first 10 days of differentiation, whereas endogenous mouse PAI-1 is not expressed (**Fig. 5A and B**, blue curves), significant amounts of exogenous human PAI-1 protein are produced. Activity of the exogenous human PAI-1 protein was verified on supernatants at day 3 using a PAI-1 activity assay. No activity was found in untreated cells, whereas a strong human PAI-1 activity was detected in doxycycline treated cells (data not shown), demonstrating that the secreted exogenous protein was functional. We then evaluated the turnover of Dox-induced human PAI-1 by quantifying by ELISA assay the remaining protein at different time after doxycycline removal (**Fig. 5C**). ESCs were treated with doxycycline either all the time or between days 0 to 3 and hPAI-1 protein was analyzed until day 7. Starting from a plateau at day 3, a similar PAI-1 protein amount is found at day 4, 50% is found at day 5 and 25% at day 7. These results suggest that the half-life of hPAI-1 protein in our cells is around 48 hrs.

### PAI-1 Overexpression in A2lox.cre ESC Clone3 Decreases ESC Adipogenesis

Doxycycline was applied during the differentiation of A2lox.cre ESC clone3 in four different conditions: i) all the time of differentiation, from days 0 to 24 **[Dox]**, ii) only from days 0 to 3 **[Dox(d0–d3)]**, iii) from days 3 to 7 **[Dox(d3–d7)]**, iv) from days 7 to 24 **[Dox(d7–d24)]** (**Fig. 5D**). The **[Dox]** and **[Dox(d7–d24)]** treatments significantly decrease by 50% the expression of adipogenic markers measured at day 24: adiponectin (**Fig. 5E**), aP2 (**Fig. 5F**) and PPARgamma (**Fig. 5G**). By contrast the **[Dox(d0–d3)]** and **[Dox(d3–d7)]** treatments have no effect. These regulations of adipocyte formation are also detected after Oil redO staining of the cultures (**Fig. 5H**).

### uPA Inhibition or PAI-1 Overexpression Modulates Successively Positively then Negatively ESC Skeletal Myogenesis

**Figure 6:**
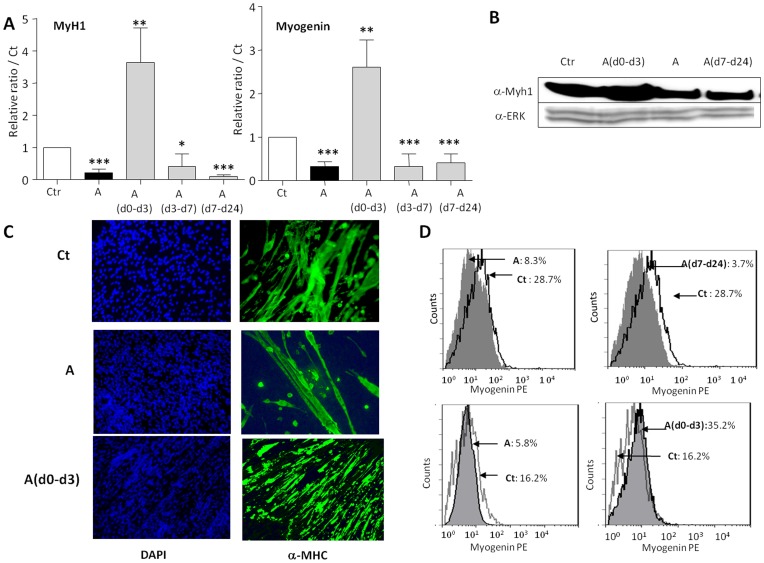
inhibition of uPA by amiloride treatments regulates ESC skeletal myogenesis. EBs from wild type CGR8 ES cells were induced to differentiate to skeletal myotube and treated or not by 100 µM amiloride for different period of time as in figure 4. (**A**) mRNAs were extracted and analyzed by real time RT-PCR for the expression of the skeletal myogenic markers MyH1 (left panel) and Myogenin (right panel). Results are expressed in arbitrary units, with the values of untreated CGR8 at day 24 taken as 1, and are the means ± S.E.M. of at least 3 independent experiments. Significance to Ct values is given as: *P<0.05, **P<0.01 and ***P<0.001. (**B**) MyH1 protein expression at day 24 of differentiation of CGR8 cells treated or not by amiloride, as indicated, was analyzed by Western blotting with anti-MyH1 (**α-MyH1**) antibodies. Membranes were reprobed with **α-ERK** antibodies as loading control. (**C**) Myotube formation of CGR8 treated or not by amiloride, as indicated, was analyzed by immunofluorescence experiments with anti-MyH1 antibodies (right panels) and nuclei were counterstained with DAPI (left panels), representative field are shown. (**D**) Differentiated cells at day 24 were analyzed for Myogenin expression by flow cytometry, two different experiments are shown, controls giving either 28.7% (upper panels) or 16.2% (lower panels) myogenin positive cells.

In parallel experiments, we investigated the role of the PA system in ESC skeletal myogenesis. The amiloride uPA inhibitor was applied on CGR8 ESCs in the same conditions as for the adipogenesis study (**Fig. 4A**). The **[A]** and **[A(d7–d24)]** amiloride treatments of CGR8 cells strongly decrease the expression of MyH1 and Myogenin skeletal muscle markers measured at day 24 (**Fig. 6A**). Although to a lesser extent, the **[A(d3–d7)]** treatment also inhibits skeletal myogenesis. Surprisingly, we found that the **[A(d0–d3)]** treatment of CGR8 cells leads to a 3.6 fold increase in expression of the MyH1 myogenic marker, and a 2.6 fold increase in Myogenin expression (**Fig. 6A**). Similar results are obtained by studying muscle proteins by western blot analysis (**Fig. 6B**), immunofluorescence experiments with anti-Myosin Heavy Chain1 antibodies (**Fig. 6C**), and flow cytometry with anti-myogenin antibodies (**Fig. 6D**).

**Figure 7:**
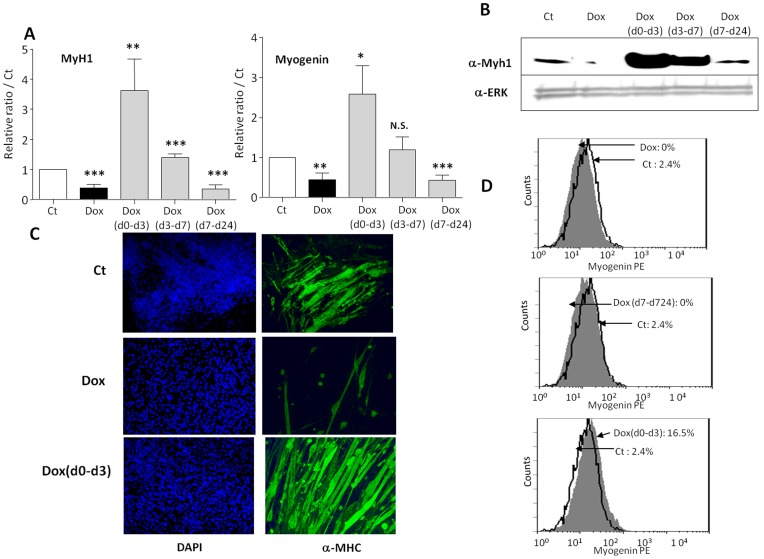
human PAI-1 expression regulates ESC skeletal myogenesis of A2lox.cre mESC clone3 after doxycycline induction. EBs from A2lox.cre mESC clone3 cells were induced to differentiate to skeletal myotube and treated or not by doxycycline for different period of time as in figure 5. (**A**) mRNAs were extracted and analyzed by real time RT-PCR for the expression of the skeletal myogenic markers MyH1 (left panel) and Myogenin (right panel). Results are expressed in arbitrary units, with the values of untreated A2lox.cre mESC clone3 cells at day 24 taken as 1, and are the means ± S.E.M. of at least 3 independent experiments. Significance is given as: *P<0.05, **P<0.01 and ***P<0.001. (**B**) MyH1 protein expression at day 24 of differentiation of A2lox.cre mESC clone3 cells treated or not by doxyxcycline, as indicated, was analyzed by Western blotting with anti-MyH1 (**α-MyH1**) antibodies. Membranes were reprobed with **α-ERK** antibodies as loading control. (**C)** Myotube formation of A2lox.cre mESC clone3 cells treated or not by doxycycline, as indicated, was analyzed by immunofluorescence experiments with anti-MyH1 antibodies (right panels) and counterstained with DAPI (left panels), representative fields are shown. (**D**) Differentiated cells at day 24 were analyzed for Myogenin expression by flow cytometry.

The effects of human PAI-1 overexpression on skeletal myogenesis of A2lox.cre ESC clone3 were investigated by applying doxycycline in the same conditions as for the adipogenesis study (**Fig. 5D**). Although less effective than in CGR8 ESCs, myogenesis is readily detectable in A2lox.cre ESCs and in its derivative clone3. We found results that parallel the effects of amiloride on CGR8 cells: **[Dox]** and **[Dox(d7–d24)]** treatments inhibit myogenesis, whereas **[Dox(d0–d3)]** activates it (**Fig. 7**). The **[Dox(d3–d7)]** condition gives a modest activation of MyH1 and no effect on Myogenin expression (**Fig. 7A**).

Interfering with the PA system does neither affect neurogenesis nor the other mesodermal ESC differentiations. Amiloride treatments have no effect on neurogenesis (**Data S6A**) and cardiomyogenesis (**Data S6B**) and doxycycline-induced overexpression of human PAI-1 does not interfere with neurogenesis (**Data S6C**), cardiomyogenesis (**Data S6D,E**), endothelial (**Data S6F, G, H**) and smooth muscle (**Data S6I**) differentiations of A2lox.cre mESC clone3. Particularly, interfering with the PA system during the d0–d3 critical period did not affect these differentiations.

### PAI-1^−/−^ Pluripotent Cells are Prone to Form Adipocytes

**Figure 8:**
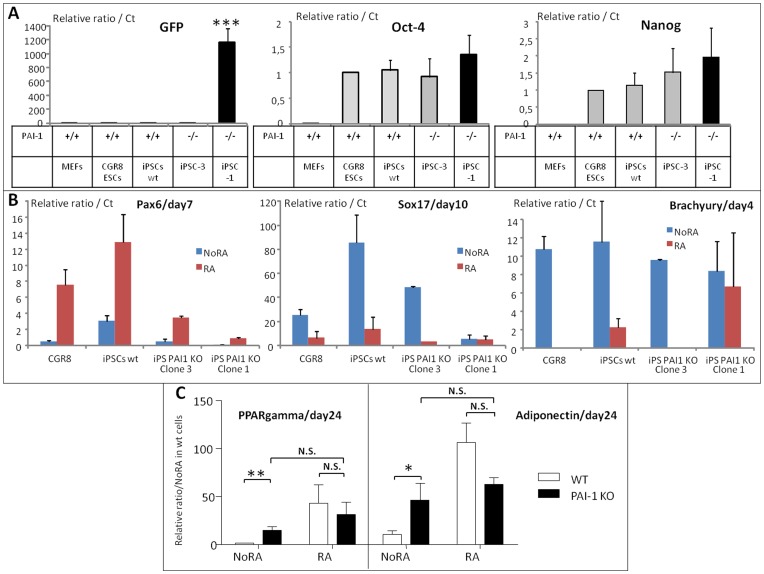
characterization and adipogenic capacities of PAI-1^−/−^ pluripotent cells. (**A**) In comparison to primary MEFs and wt CGR8 ESCs, the degree of reprogrammation of wt and PAI-1 KO, clones 1 and 3, iPSCs was characterized by the mRNA levels of Oct4 and Nanog genes and by the extinction of the expression of GFP gene. Results are expressed in arbitrary units, with the values of CGR8 mESCs taken as 1, and are the means ± S.E.M. of at least 3 independent experiments. Significance is given as: *P<0.05, **P<0.01 and ***P<0.001. (**B**) Characterization, during the differentiation process of iPSCs, of the expression of ESC commitment master genes. Retinoic acid-treated (RA) or not (NoRA) EBs from wild type CGR8 ESCs and iPSC clones were induced to differentiate. mRNAs were extracted at various time, as indicated, and analyzed by real time RT-PCR for the expression of Pax6, Sox17 and Brachyury. Results are expressed in arbitrary units, with the values of wild type CGR8 taken as 1, and are the means ± S.E.M. of at least 3 independent experiments. (**C**) Retinoic acid-treated (**RA**) or not (**NoRA**) EBs from either wild type (**WT**) pluripotent cells or PAI-1^−/−^ iPSCs (**PAI-1 KO**) were induced to differentiate to adipocytes. mRNAs were extracted and analyzed by real time RT-PCR for the expression of the adipogenic markers PPARgamma and adiponectin. Results are expressed in arbitrary units, with the values of WT NoRA condition at day 24 taken as 1, and are the means ± S.E.M. of at least 3 independent experiments. Significance is given as: N.S. non significant, *P<0.05, **P<0.01 and ***P<0.001.

To investigate the effect of the absence of PAI-1 during ESC differentiation we isolated and characterized pluripotent iPSCs from PAI-1^−/−^ mouse embryo fibroblasts (MEFs). Control MEFs and MEFs knock-out for the PAI-1 gene [27] were transduced with the reprogramming virus encoding the Klf4, Oct4, Sox2 and c-Myc transcription factors together with GFP in a single polycistronic transcript [28]. Several colonies presenting an ESC-like phenotype (see an example in **Data S7A**) were cloned and characterized for: i) mRNA and protein expression of the Nanog and Oct4 pluripotent factors, which should be comparable to ESC; ii) transgene extinction of gene expression -measured by GFP expression and by specific primers-, which signs complete epigenetic reprogrammation; and iii) *in vitro* and *in vivo* pluripotent properties. Among the pluripotent factors, Nanog is not transduced by the reprogramming vector; therefore its *de novo* expression signs a reprogrammation process. As expected, the analyzed colonies reached various degrees of reprogrammation; only some of them are completely reprogrammed. An example of this characterization is given in **Fig. 8A** and **Data S7, S8, S9**. To confirm *in vivo* their pluripotent potential, ESCs, fully reprogrammed control PAI-1^+/+^ and PAI-1 KO iPSCs (clone 3) were injected subcutaneously into nude mice. Four animals were injected for each cell line and tumours developed after four weeks in all animals; sections of the different teratomas were histologically analyzed. All teratomas presented differentiated tissues deriving from the three embryonic layers (**Data S10**).

We then chose to compare the *in vitro* differentiation potential of both fully (clone 3) and partially (clone 1) reprogrammed PAI-1 KO iPSC clones to wild-type ESCs and fully reprogrammed control PAI-1^+/+^ cells. We first checked, during the differentiation process, the expression of known master genes of the ESC commitment towards the embryonic cell lineages. We analyzed the expression of Pax6 for the ectoderm (**Fig. 8B**), Sox17 for the endoderm (**Fig. 8B**), Brachyury (**Fig. 8B**) Tbx1 and Mesp1 (**Data S11**) for the mesoderm, and FoxA2 for the mesendoderm (**Data S11**). Upon induction of ESC differentiation, the expression of these genes is characterized by early peaks, variable between days 4 to 10, which are regulated by RA. As expected, the iPSCs^+/+^ gives an expression profile of these genes very similar to the wild type CGR8 ESC control. Likewise, the partially reprogrammed iPSC clone 1 gives aberrant level of expression and RA responses in each case, demonstrating that its incomplete reprogrammation interferes with its differentiation capacities. Interestingly, the fully reprogrammed iPS PAI-1 KO clone 3 gives in majority similar expression as wt ESCs and iPSCs, except for the ectoderm Pax6 and the mesoderm Tbx1 master genes.

We found no difference in the capacities of PAI-1^−/−^ iPSCs to form cardiomyocytes and myotubes (data not shown). By contrast, at the opposite of control wild-type cells which necessitate an early retinoic acid treatment to form adipocytes by day 24, the PAI-1^−/−^ iPSCs form adipocytes spontaneously and do not respond to the retinoic acid treatment (**Fig. 8C**).

## Discussion

Regulation of proteolytic activity generated by the plasminogen activation system is involved in many physiopathological processes; in this study we demonstrate that it controls several distinct and successive steps of the *in vitro* differentiation of embryonic stem cells. We found that, during the terminal differentiation of ESCs, either uPA inhibition or PAI-1 overexpression strongly and concomitantly decrease adipogenesis and skeletal myogenesis. Likewise, inhibitory effects were observed on adipogenesis when interfering with the PA system during the early commitment of ESCs. By contrast, skeletal myogenesis is stimulated by an early uPA inhibition or PAI-1 overexpression. Although we cannot exclude that tPA plays a role in ESC differentiation, we reproduced most of the PAI-1 overexpression effects by inhibiting uPA with amiloride, suggesting that, in our experimental model, PAI-1 is mainly acting via inhibition of uPA. Interestingly these regulations are specific to the adipogenic and skeletal myogenic lineages; indeed neither endothelial cell formation, smooth muscle myogenesis, cardiomyogenesis nor neurogenesis are affected.

The decrease of adipogenesis resulting from inhibition of uPA or from PAI-1 overexpression, during the terminal differentiation of ESCs is in agreement with the previous *in vitro* roles described for uPA and PAI-1 in adipocyte differentiation [9]. It is also consistent with *in vivo* data showing that PAI-1^−/−^ mice are prone to obesity [27]. Between days 0 to 7 of the differentiation protocol of ESCs, embryoid bodies recapitulate the formation of the three embryonic germ layers. Interestingly, during this period, whereas endogenous PAI-1 is not expressed, maximum uPA enzymatic activity is detected. During this early period, we found that uPA inhibition decreases adipogenesis. To explain these inhibitory effects one could hypothesize that the PA system regulates ESC differentiation by interfering with extracellular matrix deposition. However our preliminary results show that even if the PA system modulates collagen-1 deposition, there is no direct correlation between this effect and the PA-mediated effects on ESC adipogenesis. Interestingly, the PA system has also been implicated in the activation of three major intracellular transduction pathways: ERK, PI3K/PKB and JAK/STAT pathways [34], themselves involved in adipogenesis. One can hypothesize that one or several of these pathways are implicated in ESC differentiation in response to the PA system. It has been already shown that PAI-1 prevents the cooperation of αvβ3 integrin with insulin signaling in NIH3T3 fibroblasts, resulting in a decrease in insulin-induced PKB phosphorylation [35]. Since, insulin is required for the terminal step of differentiation of ESC towards adipocytes [30], this pathway could be involved in the effects that we observed in response to PA interference during ESC adipogenesis. Whether the early (d0–d7) and late (d7–d24) effects are regulated by common or different molecular mechanisms requires further studies. However, it is very likely that they correspond to the specific targeting of successive early precursors of the adipocyte lineage. Indeed, despite the fact that such precursors have not be precisely identified nor characterized, we know that PPARgamma, the master gene of terminal adipocyte differentiation, is not present before day 15 of ESC differentiation [30]. Therefore we believe that the PA modulation of the ESC experimental model could constitute a powerful tool to characterize the early precursors of the adipogenic lineage. For example, our results suggest that during the early period, one can specifically target the adipocyte lineage by an amiloride treatment, potentially allowing the identification of important adipogenic genes differentially regulated during this precise timing.

Though PAI-1 ectopic overexpression during ESC terminal adipocyte differentiation gave similar inhibition than amiloride treatment, intriguingly, early (d0–d7) overexpression during differentiation has no effect on adipogenesis. To explain this lack of effect one can hypothesize that, during the early steps of ESC commitment, PAI-1 protein would lead to both adipogenic inhibitory effects, via uPA enzymatic inhibition, and adipogenic stimulatory effects, independently of uPA; resulting in the net absence of effect on adipogenesis. Additional works are necessary to further explore this hypothesis.

Finally the analysis of PAI-1^−/−^ iPSCs constituted a third converging argument to demonstrate the implication of the PA system in ESC adipogenesis. Indeed, these cells are prone to form adipocytes spontaneously and, contrarily to wild type cells, do not respond anymore to the retinoic acid treatment. Furthermore, these results fit with the observation that knock-out PAI-1 mice are prone to obesity [27].

Regarding skeletal myogenesis and similarly to adipogenesis, we found a decrease of differentiation resulting from uPA inhibition or PAI-1 ectopic overexpression during the terminal differentiation of ESCs. These results are in agreement with the previous roles described for uPA and PAI-1 in *in vitro* muscle cell differentiation [15] and *in vivo* skeletal muscle regeneration [16], [17]. Surprisingly, EB treated between days 0 to 3 presented a significant increase in skeletal myogenesis compared to untreated cells. The two approaches, uPA inhibition or PAI-1 overexpression, gave similar results. Because of the low expression level of the proteases during this period, we did not expect an effect by interfering with the PA. However, our results are consistent with the fact that a robust enzymatic activity of uPA is effectively detected at day 3. During this period uPA activity seems to specifically repress skeletal myogenesis, demonstrating that the commitment towards this particular mesodermal lineage can be modulated very early during differentiation. Whether interfering with the PA system at that step, targets directly or indirectly putative skeletal myogenic precursors needs to be investigated. Interestingly, the expression of the transcription factors Pax3, Pax7 and MyoD that are known to be necessary during the early steps of myogenesis, were undetectable at that time, appearing only after day 14 of the differentiation protocol (data not shown); suggesting that our effect targets earlier myogenic precursors.

Therefore, by pharmaceutical and genetic experiments we found that early uPA inhibition, between days 0 to 3 of differentiation, augments ESC myogenesis. To our knowledge, this is the first time that such augmentation of *in vitro* myotube formation in the mouse ESC model is described. Other ESC myogenic stimulations described in the literature are limited to the formation myogenic precursors, as for example, the treatment of EBs with low doses of retinoic acid [36]. Since full skeletal myogenesis from ESCs is a complex, long and difficult process to reproduce in culture, especially within the human ESC model, we believe that our results represent an important contribution in order to fully differentiate ESCs towards this lineage.

Our results show that the PA system is capable to specifically and concomitantly regulate two complex multi-steps ESC lineages, adipocyte and skeletal muscle. Particularly, the very early opposite regulation evidenced here, between days 0 to 3, could suggests that, during that period, uPA activity induces a switch augmenting, on the one hand, adipogenesis and inhibiting, on the other hand, skeletal myogenesis. This is reminiscent to a comparable switch regulation between neurogenesis and cardiomyogenesis induced by the p38MAPK that we already described in this model [24]. To better understand the molecular mechanisms involved in such switch, we need to identify the cellular precursors affected by these processes.

Importantly, our results demonstrate that the simple interference with the modulation of the extracellular matrix during ESC differentiation is capable to commit ESC towards specific cell lineages. These regulations taking place at the cellular membrane and being reversible can be of great interest for the use of committed ESCs in cellular therapy.

## Supporting Information

Data S1 **Immunocytochemistry analysis was performed on CGR8 cells at day 24 of differentiation, using either control antibodies (upper panels) or anti-PAI-1 antibodies (lower panels).** Photographs show representative fields of differentiated cultures and arrows indicated either well differentiated adipocytes (obtained in RA condition, left panel) or skeletal myotubes (obtained in NoRA condition, right panel) stained by PAI-1.
(TIF)

Data S2 **intracellular inhibition of uPA by amiloride treatments.** Retinoic acid-treated (adipogenic conditions) or not (skeletal myogenic conditions) EBs from wild type CGR8 ES cells were induced to differentiate and treated or not by 100 µM amiloride for different period of time, as indicated. Intracellular mouse activated uPA and the inhibitory effects of amiloride treatments were quantified by ELISA assay. Mean values of at least three independent experiments are given.
(TIF)

Data S3 **effects of amiloride treatments on collagen-1 secreted by ESCs induced to differentiate in adipogenic conditions.** Retinoic acid-treated (adipogenic conditions) EBs from wild type CGR8 ES cells were induced to differentiate and treated or not by 100 µM amiloride for different period of time, as indicated. At day 12 (left panels) or 24 (right panels) cells were removed and the extracellular matrix was examined by immunofluorescence experiments using anti-collagen 1 antibodies. Photographs show representative fields.
(TIF)

Data S4 **effects of amiloride treatments on collagen-1 secreted by ESCs induced to differentiate in myogenic conditions.** Non retinoic acid-treated (myogenic conditions) EBs from wild type CGR8 ES cells were induced to differentiate and treated or not by 100 µM amiloride for different period of time, as indicated. At day 12 (left panels) or 24 (right panels) cells were removed and the extracellular matrix was examined by immunofluorescence experiments using anti-collagen 1 antibodies. Photographs show representative fields.
(TIF)

Data S5 **mPAI-1 and uPA expression in wild type A2lox.cre mESCs.** (**A** and **B**) Retinoic acid-treated (**RA**) or not (**NoRA**) EBs from A2lox.cre mESCs were induced to differentiate and analyzed at various time, as indicated, between days 0 (**d0**) to 24 (**d24**). Endogenous mouse (mPAI-1) PAI-1 protein expressions in conditioned medium (**A**) and cell lysates (**B**) were quantified by ELISA technique at various time, as indicated, between days 0 to 24. Values are given in ng of PAI-1 amounts and expressed as means of at least three independent experiments ± S.E.M. (**C** and **D**) Retinoic acid-treated (RA) or not (NoRA) EBs from wild type CGR8 and A2lox.cre mESCs cells were induced to differentiate and analyzed at day d14 (**C**) and day 24 (**D**). mRNAs were extracted and analyzed by real time RT-PCR for the expression of uPA. Results are expressed in arbitrary units, with the values of wild type CGR8 taken as 1, and are the means ± S.E.M. of at least 3 independent experiments.
(TIF)

Data S6 **amiloride treatments do not interfere with either neurogenesis or cardiomyogenesis of CGR8 ESCs and doxycycline-induced human PAI-1 expression does not interfere with either neurogenesis, cardiomyogenesis, endothelial or smooth muscle differentiations of A2lox.cre ESC clone3.** (**A** and **B**) Retinoic acid-treated (**A**) or not (**B**) EBs from wild type CGR8 ESCs were induced to differentiate and treated or not by 100 µM amiloride for different period of time: from days 0 to 12 **[A]**, from days 0 to 3 **[A(d0–d3)]**, from days 3 to 7 **[A(d3–d7)]**, from days 7 to 12 **[A(d7–d12)]**. mRNAs were extracted and analyzed by real time RT-PCR for the expression of the neuronal marker MAP2 (**A**) and for the cardiomyocyte marker GATA4 (**B**). Results are expressed in arbitrary units, with the values of untreated CGR8 at day 12 taken as 1, and are the means ± S.E.M. of at least 3 independent experiments. (**C**, **D** and **E**) Retinoic acid-treated (**C**) or not (**D** and **E**) EBs from A2lox.cre mESC clone3 cells were induced to differentiate and treated or not by doxycycline for different period of time: from days 0 to 12 **[Dox]**, from days 0 to 7 **[Dox(d0–d7)]**, from days 7 to 12 **[Dox(d7–d12)]**. mRNAs were extracted and analyzed by real time RT-PCR for the expression of the neuronal marker MAP2 (**C**) and for the cardiomyocyte markers GATA4 (**D**) and troponinT (**E**). Results are expressed in arbitrary units, with the values of untreated A2lox.cre mESC clone3 cells at day 12 taken as 1, and are the means ± S.E.M. of at least 3 independent experiments. (**F,** **G, H** and **I**) EBs from A2lox.cre mESC clone3 cells were induced to differentiate and treated **(Dox)** or not **(Ct)** by doxycycline from days 0 to 12 and analyzed at various time, as indicated, between days 0 to 12. mRNAs were extracted and analyzed by real time RT-PCR for the expression of the endothelial markers Flk1 (**F**), von Willebrand Factor (**G**) and PECAM (**H**) for the smooth muscle marker α-SMA (**I**). Results are expressed in arbitrary units, with the values of untreated A2lox.cre mESC clone3 cells at day 3 taken as 1, and are the means ± S.E.M. of at least 3 independent experiments.
(TIF)

Data S7 **characterization of PAI-1^+/+^ and ^−/−^ iPSCs: phenotype and transgene expression.** (**A**) Photographs of cell cultures at the undifferentiated state of: CGR8 wt ESCs (upper left), iPSC wt clone (upper right), iPSC PAI-1 KO clone 3 (lower left), iPSC PAI-1 KO clone 1 (lower right). Several typical packed colonies with an ESC phenotype (red arrows) are visible on each photograph. Pluripotent cells were grown on top of a feeder layer which is composed of mitomycine-treated primary mouse embryo fibroblasts (white arrows). (**B**) In comparison to primary MEFs and wt CGR8 ESCs, the degree of reprogrammation of wt and PAI-1 KO, clones 1 and 3, iPSCs was characterized by the extinction of the transgene expression. Results are expressed in arbitrary units, with the values of CGR8 mESCs taken as 1, and are the means ± S.E.M. of at least 3 independent experiments. Significance is given as: *P<0.05. (**C**) Oct4 protein expression at the undifferentiated state of CGR8 wt ESCs, iPSC wt clone, iPSC PAI-1 KO clone 3 and iPSC PAI-1 KO clone 1 was analyzed by Western blotting with anti-Oct4 **(α-Oct4**) antibodies. Membranes were reprobed with **α-ERK** antibodies as loading control.
(TIF)

Data S8 **characterization of PAI-1^+/+^ and ^−/−^ iPSCs: Nanog and Oct4 immunoflurescence.** Immunofluorescence staining of cell cultures at the undifferentiated state of: CGR8 wt ESCs, iPSC wt clone, iPSC PAI-1 KO clone 3 and MEFs, as indicated. Cells were labeled with anti-Nanog antibodies (upper panels) or anti-Oct4 antibodies (lower panels). Same microscope fields labeled with DAPI are shown (right panels).
(TIF)

Data S9 **characterization of PAI-1^+/+^ and ^−/−^ iPSCs: Sox2 and SSEA1 immunofluorescence.** Immunofluorescence staining of cell cultures at the undifferentiated state of: CGR8 wt ESCs, iPSC wt clone, iPSC PAI-1 KO clone 3 and MEFs, as indicated. Cells were labeled with anti-Sox2 antibodies (upper panels) or anti-SSEA1 antibodies (lower panels). Same microscope fields labeled with DAPI are shown (right panels).
(TIF)

Data S10 **histological characterization of PAI-1^+/+^ and ^−/−^ iPSC teratoma.** CGR8 wt ESCs (**A**), iPSC wt clone (**B**), and iPSC PAI-1 KO clone 3 (**C**) were injected subcutaneously into nude mice. After four weeks, sections of the different teratomas were histologically analyzed. All teratomas presented differentiated tissues deriving from the three embryonic layers. Representative fields of well-differentiated tissues are shown. Arrows indicate examples of the three germ layer derivative structures: **Cart**  =  cartilage (mesoderm derivative), **Epit**  =  ciliated epithelium (endoderm derivative), and neural structures (ectoderm derivative): neural rosettes and nerve fibers.
(TIF)

Data S11 **characterization of PAI-1^+/+^ and ^−/−^ iPSCs: in vitro differentiation.** Characterization of the expression of ESC commitment master genes during the differentiation process of iPSCs. Retinoic acid-treated (RA) or not (NoRA) EBs from wild type CGR8 ESCs and iPSC clones were induced to differentiate. mRNAs were extracted at day 10 and analyzed by real time RT-PCR for the expression of FoxA2, Mesp1 and Tbx1. Results are expressed in arbitrary units, with the values of wild type CGR8 at day 0 taken as 1, and are the means ± S.E.M. of at least 3 independent experiments.
(TIF)

Data S12 **Forward and reverse primer sequences of the various genes analyzed by real-time RT-PCR.**
(TIF)
