# Supplementary figures and images for: The Plasminogen Activation System Modulates Differently Adipogenesis and Myogenesis of Embryonic Stem Cells
Source: PLoS One. 2012 Nov 8;7(11):e49065. doi: 10.1371/journal.pone.0049065 (PMC3493518; doi:10.1371/journal.pone.0049065)

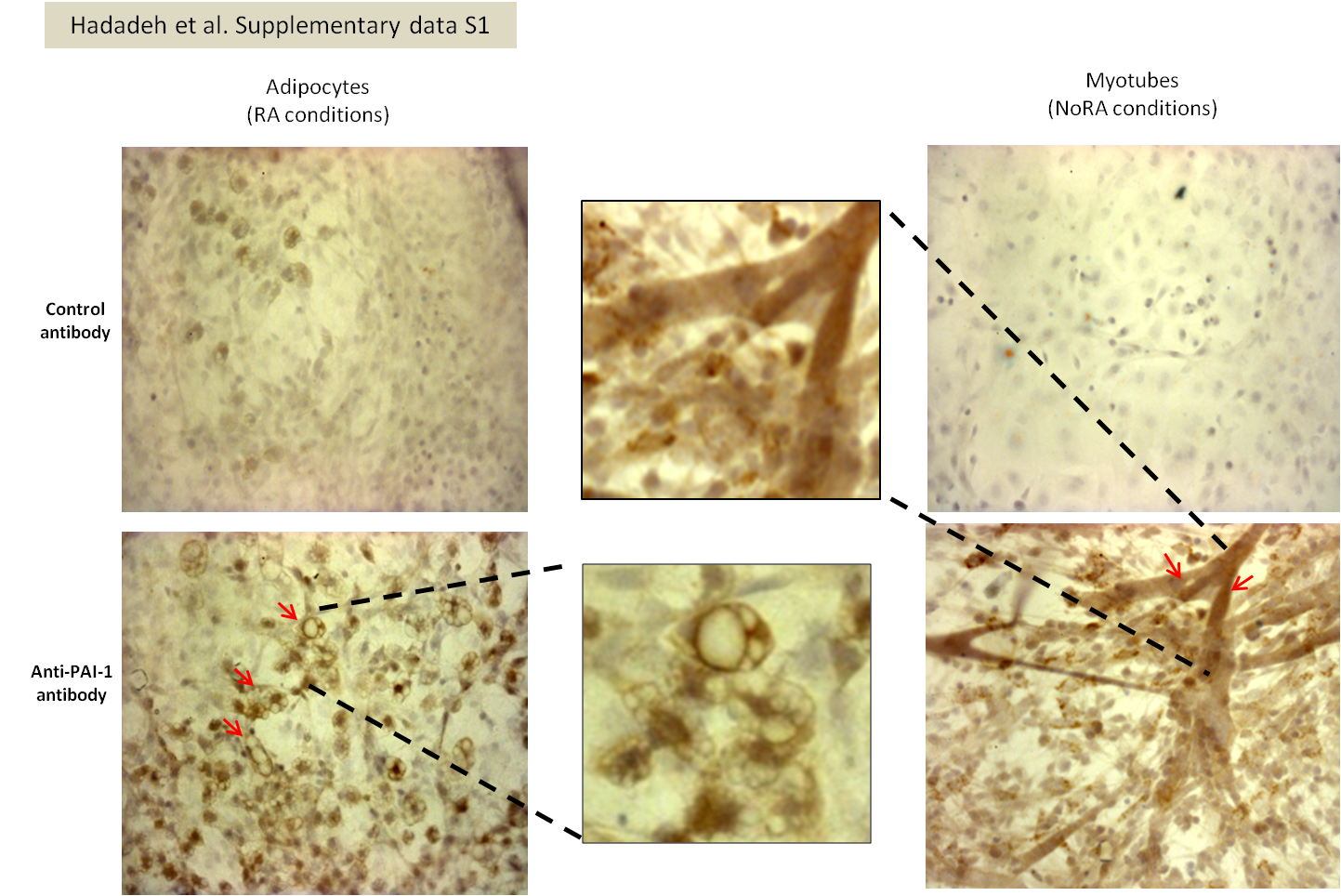

Supplement: Data S1 — Immunocytochemistry analysis was performed on CGR8 cells at day 24 of differentiation, using either control antibodies (upper panels) or anti-PAI-1 antibodies (lower panels). Photographs show representative fields of differentiated cultures and arrows indicated either well differentiated adipocytes (obtained in RA condition, left panel) or skeletal myotubes (obtained in NoRA condition, right panel) stained by PAI-1. (TIF) [file pone.0049065.s001.tif]

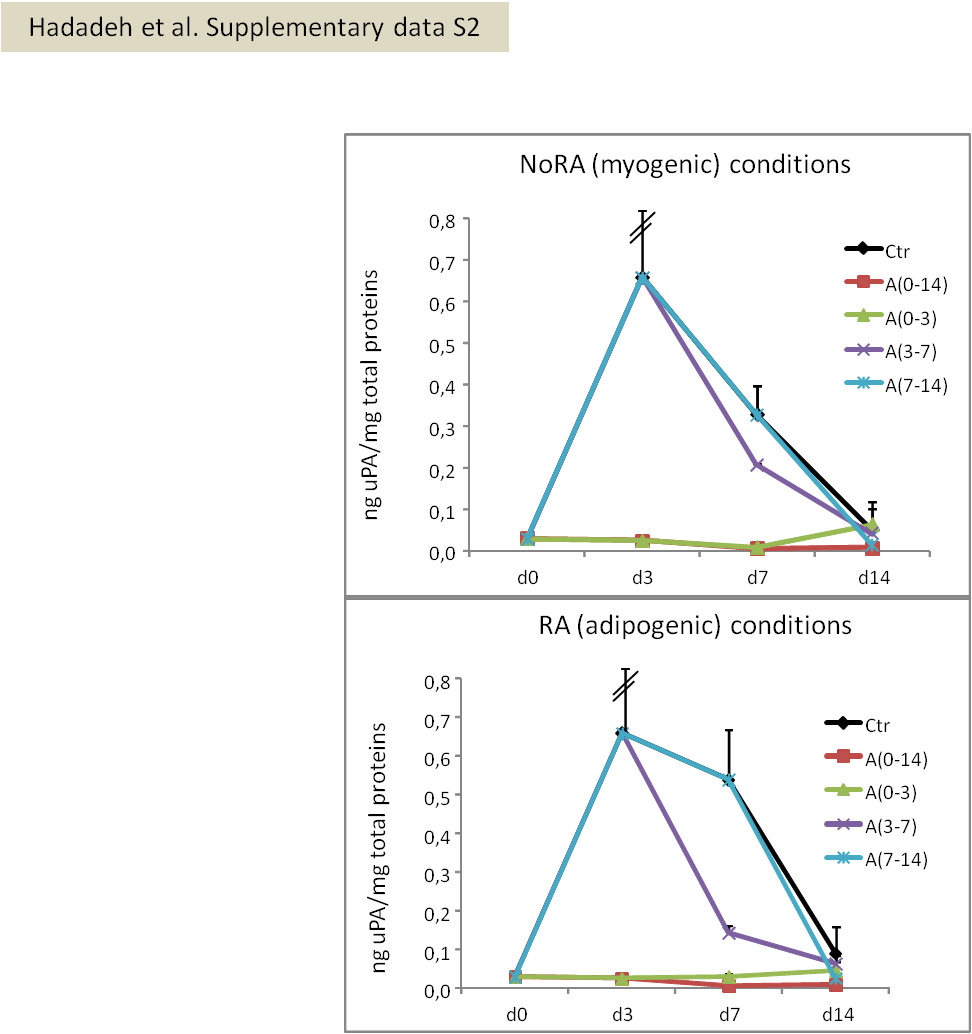

Supplement: Data S2 — intracellular inhibition of uPA by amiloride treatments. Retinoic acid-treated (adipogenic conditions) or not (skeletal myogenic conditions) EBs from wild type CGR8 ES cells were induced to differentiate and treated or not by 100 µM amiloride for different period of time, as indicated. Intracellular mouse activated uPA and the inhibitory effects of amiloride treatments were quantified by ELISA assay. Mean values of at least three independent experiments are given. (TIF) [file pone.0049065.s002.tif]

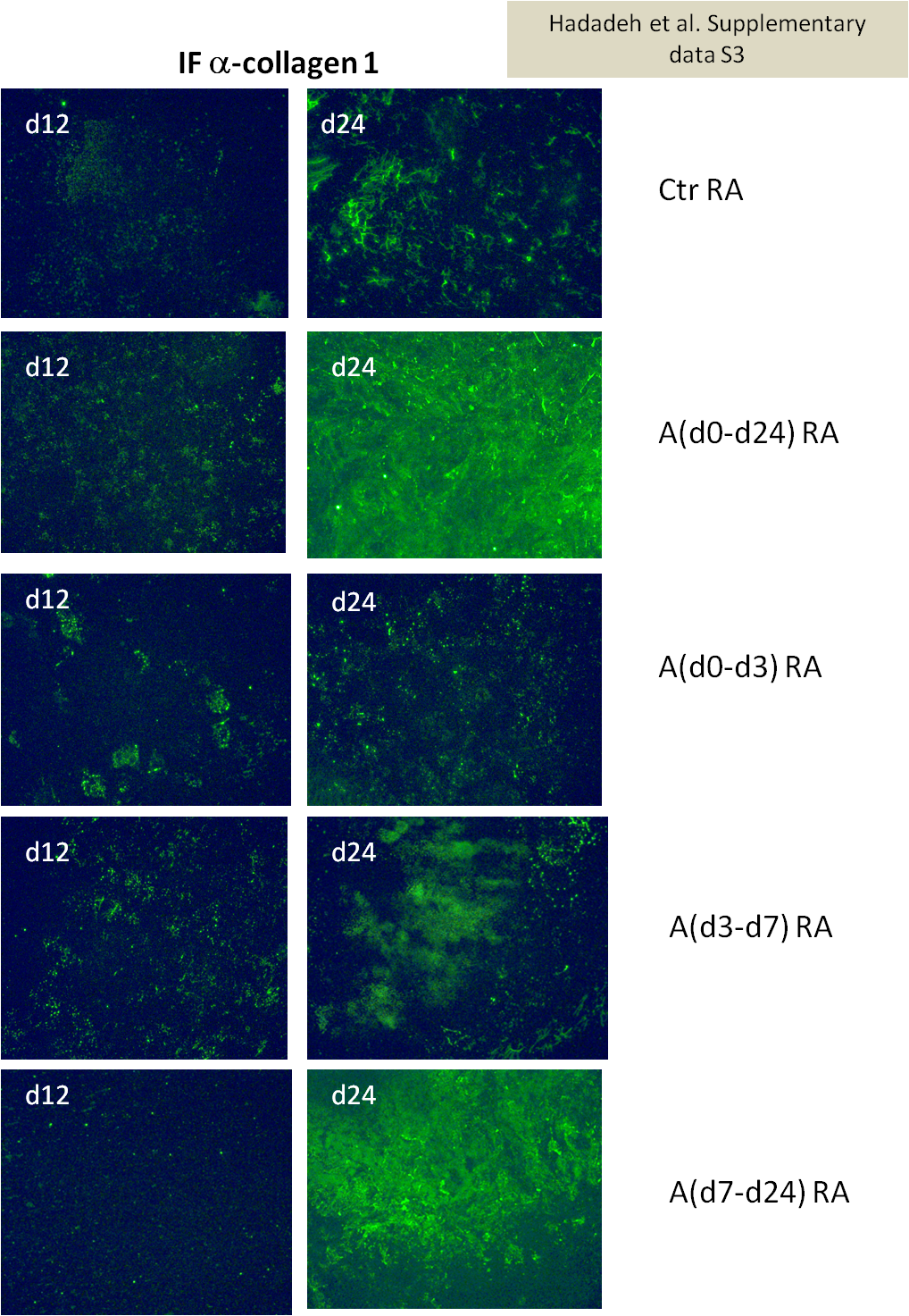

Supplement: Data S3 — effects of amiloride treatments on collagen-1 secreted by ESCs induced to differentiate in adipogenic conditions. Retinoic acid-treated (adipogenic conditions) EBs from wild type CGR8 ES cells were induced to differentiate and treated or not by 100 µM amiloride for different period of time, as indicated. At day 12 (left panels) or 24 (right panels) cells were removed and the extracellular matrix was examined by immunofluorescence experiments using anti-collagen 1 antibodies. Photographs show representative fields. (TIF) [file pone.0049065.s003.tif]

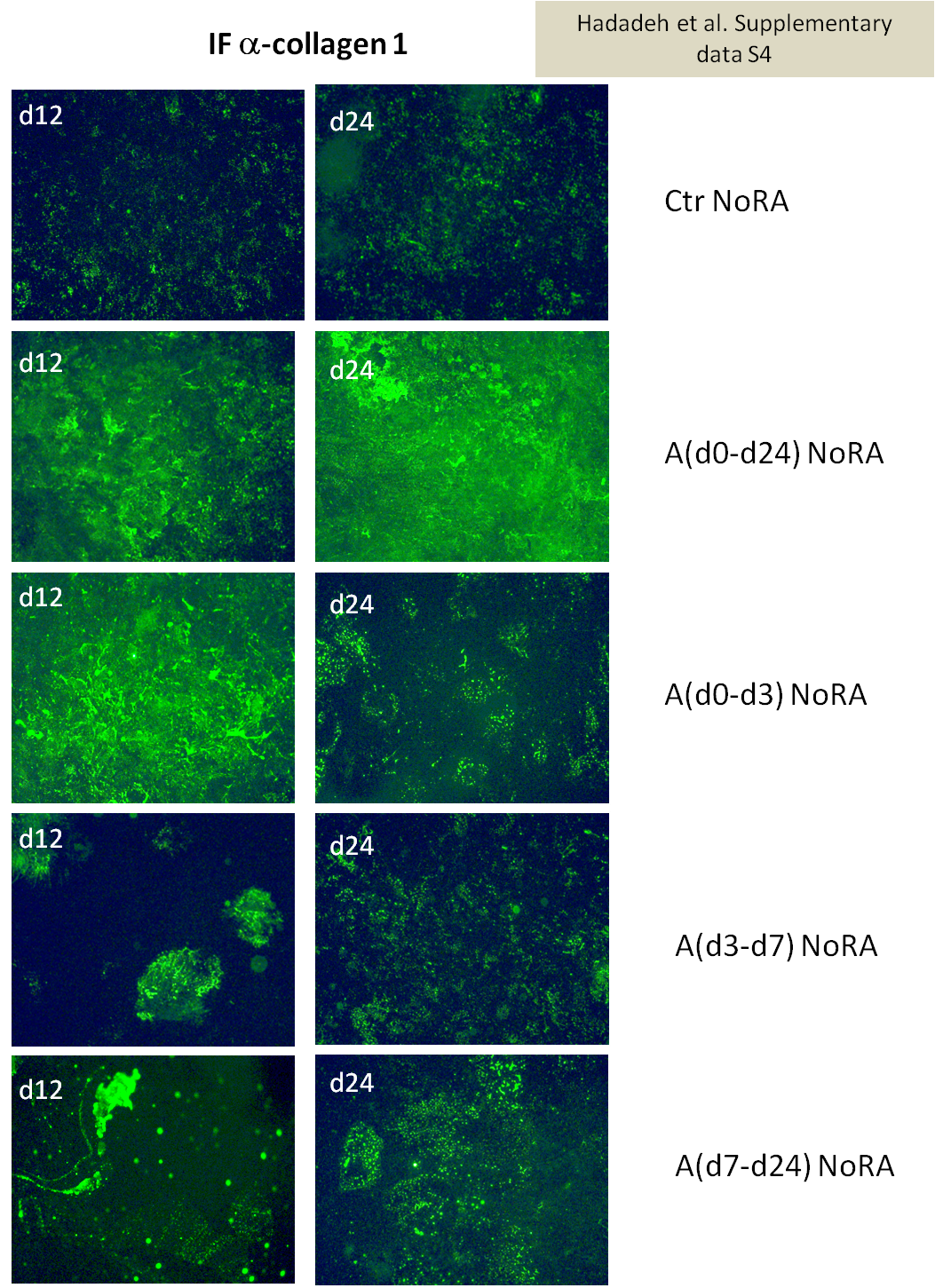

Supplement: Data S4 — effects of amiloride treatments on collagen-1 secreted by ESCs induced to differentiate in myogenic conditions. Non retinoic acid-treated (myogenic conditions) EBs from wild type CGR8 ES cells were induced to differentiate and treated or not by 100 µM amiloride for different period of time, as indicated. At day 12 (left panels) or 24 (right panels) cells were removed and the extracellular matrix was examined by immunofluorescence experiments using anti-collagen 1 antibodies. Photographs show representative fields. (TIF) [file pone.0049065.s004.tif]

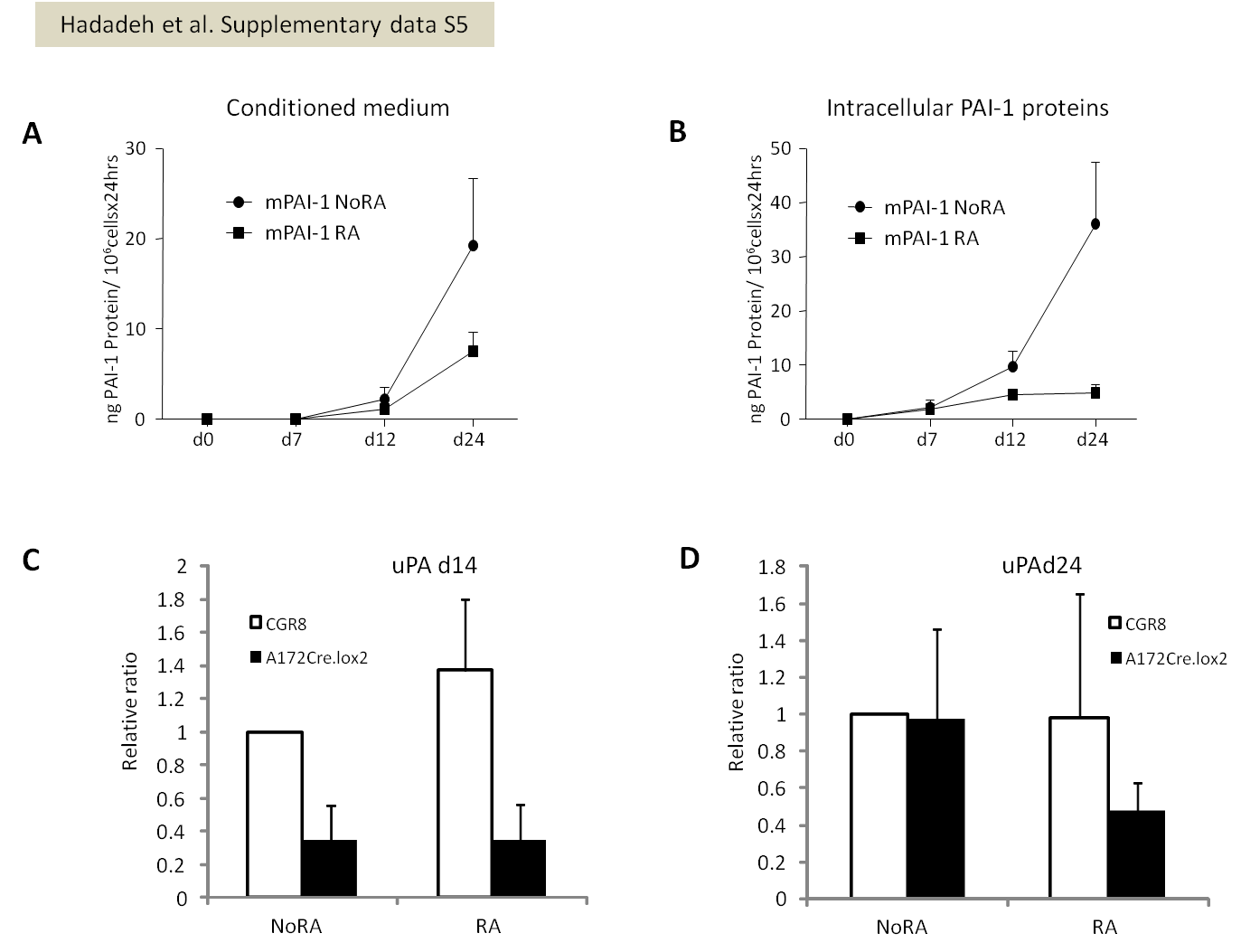

Supplement: Data S5 — mPAI-1 and uPA expression in wild type A2lox.cre mESCs. (A and B) Retinoic acid-treated (RA) or not (NoRA) EBs from A2lox.cre mESCs were induced to differentiate and analyzed at various time, as indicated, between days 0 (d0) to 24 (d24). Endogenous mouse (mPAI-1) PAI-1 protein expressions in conditioned medium (A) and cell lysates (B) were quantified by ELISA technique at various time, as indicated, between days 0 to 24. Values are given in ng of PAI-1 amounts and expressed as means of at least three independent experiments ± S.E.M. (C and D) Retinoic acid-treated (RA) or not (NoRA) EBs from wild type CGR8 and A2lox.cre mESCs cells were induced to differentiate and analyzed at day d14 (C) and day 24 (D). mRNAs were extracted and analyzed by real time RT-PCR for the expression of uPA. Results are expressed in arbitrary units, with the values of wild type CGR8 taken as 1, and are the means ± S.E.M. of at least 3 independent experiments. (TIF) [file pone.0049065.s005.tif]

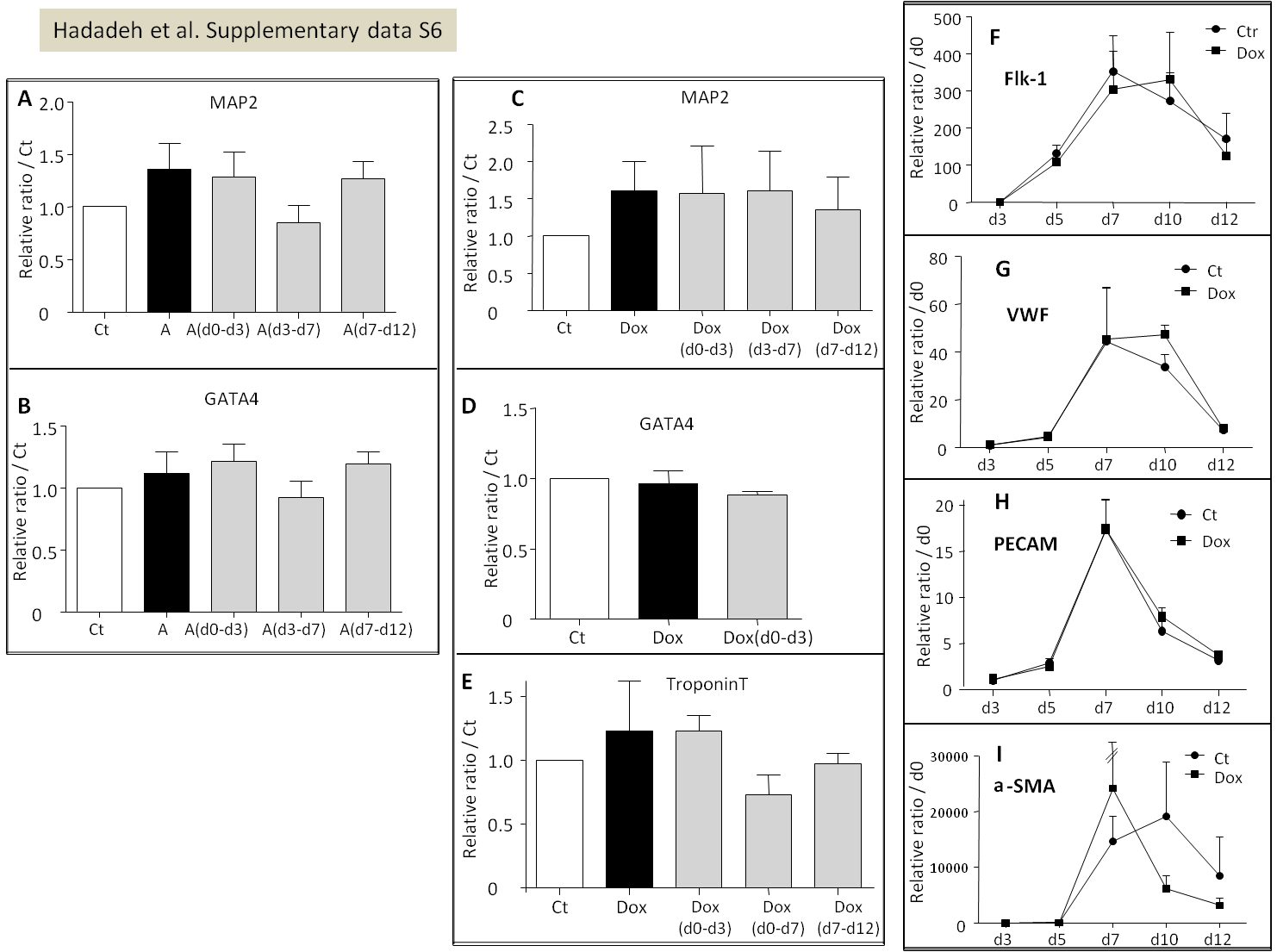

Supplement: Data S6 — amiloride treatments do not interfere with either neurogenesis or cardiomyogenesis of CGR8 ESCs and doxycycline-induced human PAI-1 expression does not interfere with either neurogenesis, cardiomyogenesis, endothelial or smooth muscle differentiations of A2lox.cre ESC clone3. (A and B) Retinoic acid-treated (A) or not (B) EBs from wild type CGR8 ESCs were induced to differentiate and treated or not by 100 µM amiloride for different period of time: from days 0 to 12 [A], from days 0 to 3 [A(d0–d3)], from days 3 to 7 [A(d3–d7)], from days 7 to 12 [A(d7–d12)]. mRNAs were extracted and analyzed by real time RT-PCR for the expression of the neuronal marker MAP2 (A) and for the cardiomyocyte marker GATA4 (B). Results are expressed in arbitrary units, with the values of untreated CGR8 at day 12 taken as 1, and are the means ± S.E.M. of at least 3 independent experiments. (C, D and E) Retinoic acid-treated (C) or not (D and E) EBs from A2lox.cre mESC clone3 cells were induced to differentiate and treated or not by doxycycline for different period of time: from days 0 to 12 [Dox], from days 0 to 7 [Dox(d0–d7)], from days 7 to 12 [Dox(d7–d12)]. mRNAs were extracted and analyzed by real time RT-PCR for the expression of the neuronal marker MAP2 (C) and for the cardiomyocyte markers GATA4 (D) and troponinT (E). Results are expressed in arbitrary units, with the values of untreated A2lox.cre mESC clone3 cells at day 12 taken as 1, and are the means ± S.E.M. of at least 3 independent experiments. (F, G, H and I) EBs from A2lox.cre mESC clone3 cells were induced to differentiate and treated (Dox) or not (Ct) by doxycycline from days 0 to 12 and analyzed at various time, as indicated, between days 0 to 12. mRNAs were extracted and analyzed by real time RT-PCR for the expression of the endothelial markers Flk1 (F), von Willebrand Factor (G) and PECAM (H) for the smooth muscle marker α-SMA (I). Results are expressed in arbitrary units, with the values of untreated A2lox.cre mESC clon [file pone.0049065.s006.tif]

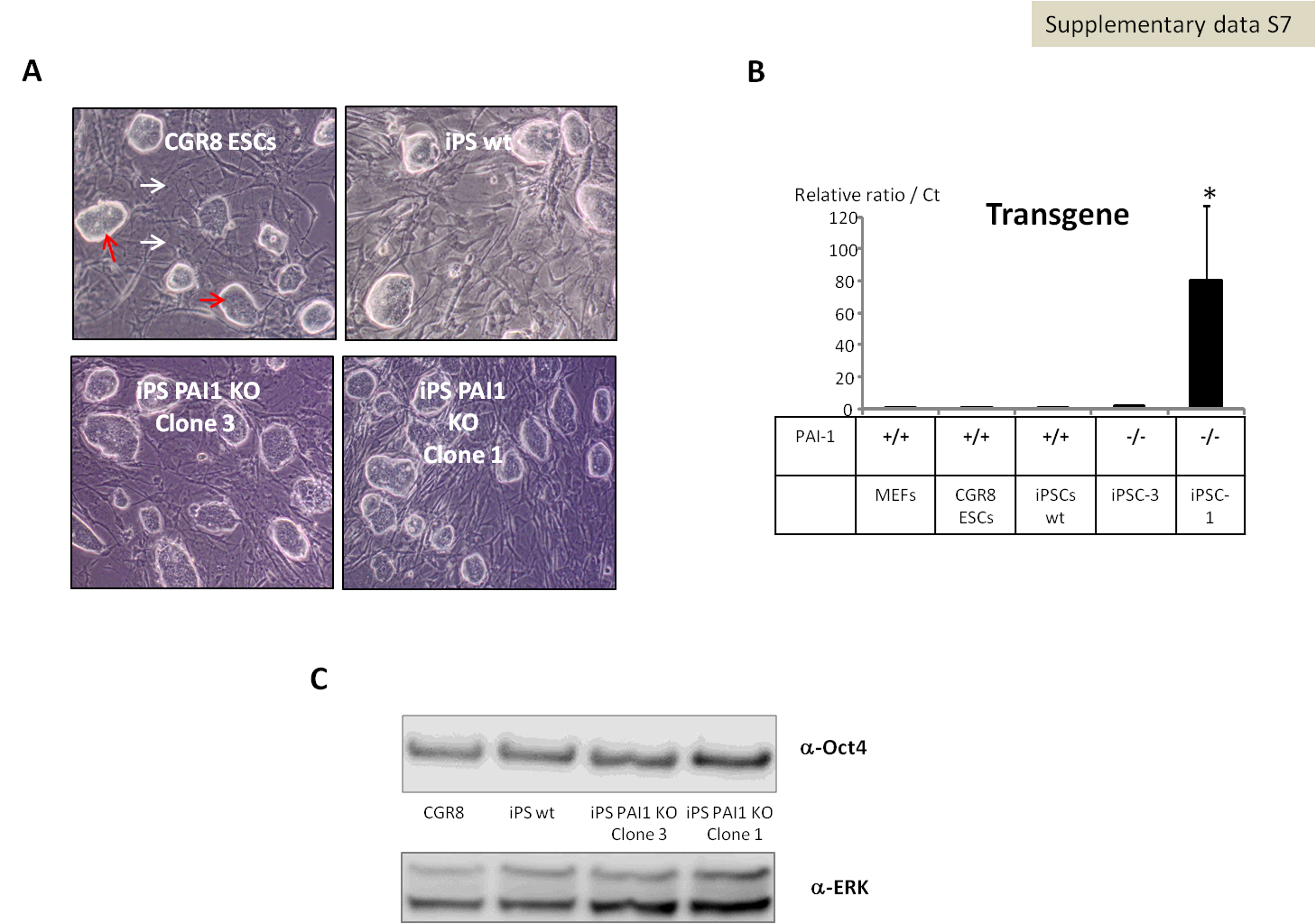

Supplement: Data S7 — characterization of PAI-1+/+ and −/− iPSCs: phenotype and transgene expression. (A) Photographs of cell cultures at the undifferentiated state of: CGR8 wt ESCs (upper left), iPSC wt clone (upper right), iPSC PAI-1 KO clone 3 (lower left), iPSC PAI-1 KO clone 1 (lower right). Several typical packed colonies with an ESC phenotype (red arrows) are visible on each photograph. Pluripotent cells were grown on top of a feeder layer which is composed of mitomycine-treated primary mouse embryo fibroblasts (white arrows). (B) In comparison to primary MEFs and wt CGR8 ESCs, the degree of reprogrammation of wt and PAI-1 KO, clones 1 and 3, iPSCs was characterized by the extinction of the transgene expression. Results are expressed in arbitrary units, with the values of CGR8 mESCs taken as 1, and are the means ± S.E.M. of at least 3 independent experiments. Significance is given as: *P<0.05. (C) Oct4 protein expression at the undifferentiated state of CGR8 wt ESCs, iPSC wt clone, iPSC PAI-1 KO clone 3 and iPSC PAI-1 KO clone 1 was analyzed by Western blotting with anti-Oct4 (α-Oct4) antibodies. Membranes were reprobed with α-ERK antibodies as loading control. (TIF) [file pone.0049065.s007.tif]

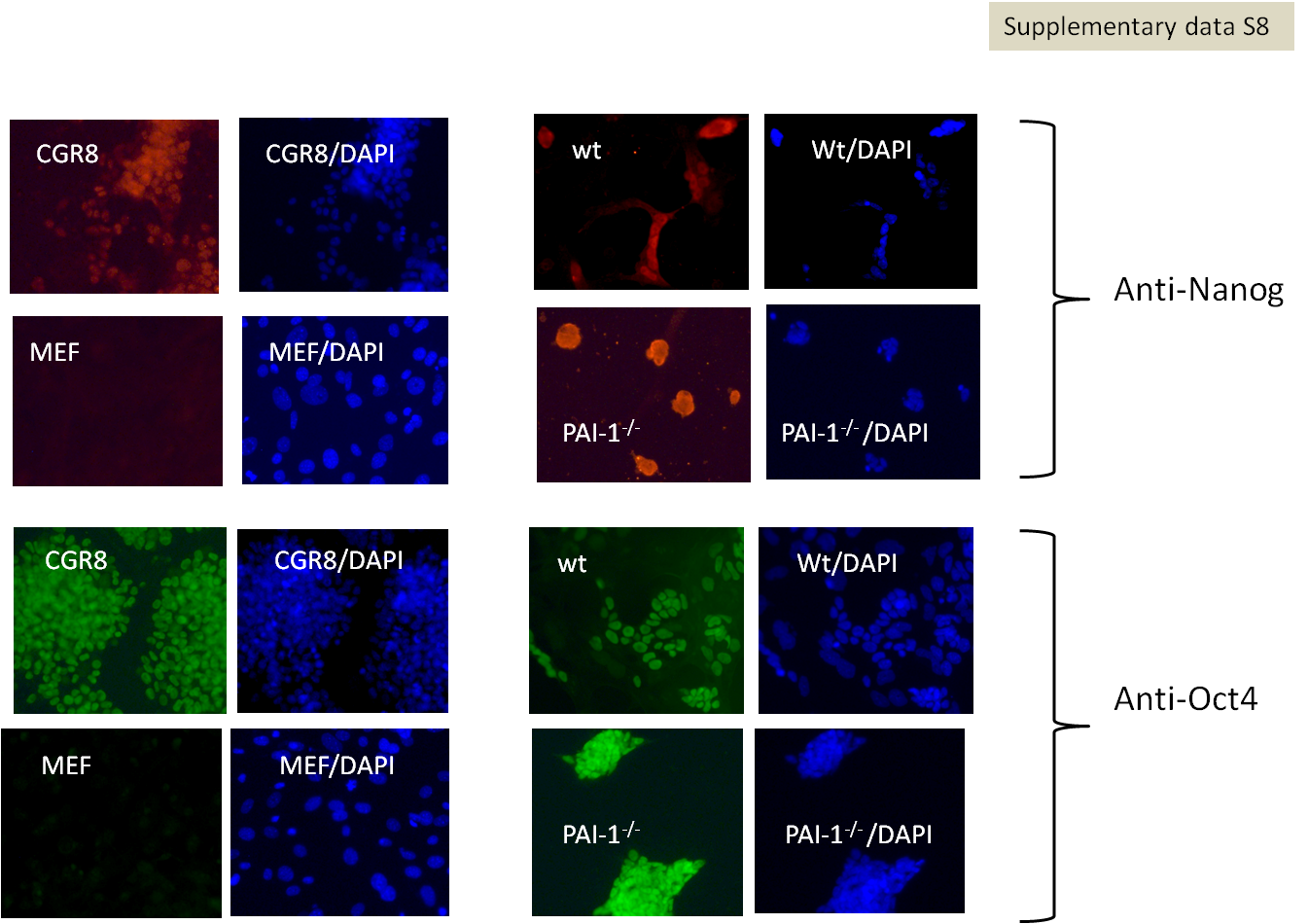

Supplement: Data S8 — characterization of PAI-1+/+ and −/− iPSCs: Nanog and Oct4 immunoflurescence. Immunofluorescence staining of cell cultures at the undifferentiated state of: CGR8 wt ESCs, iPSC wt clone, iPSC PAI-1 KO clone 3 and MEFs, as indicated. Cells were labeled with anti-Nanog antibodies (upper panels) or anti-Oct4 antibodies (lower panels). Same microscope fields labeled with DAPI are shown (right panels). (TIF) [file pone.0049065.s008.tif]

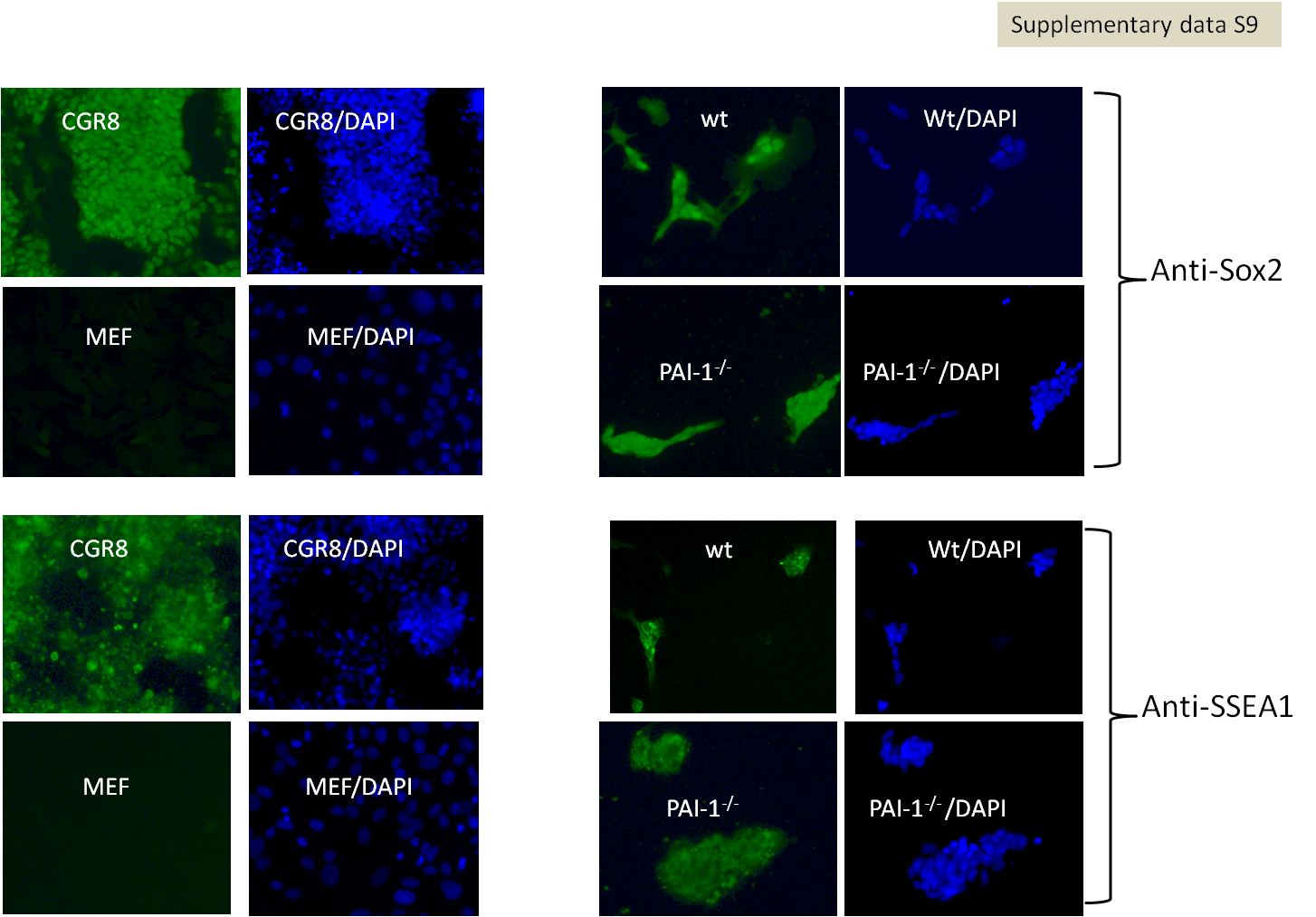

Supplement: Data S9 — characterization of PAI-1+/+ and −/− iPSCs: Sox2 and SSEA1 immunofluorescence. Immunofluorescence staining of cell cultures at the undifferentiated state of: CGR8 wt ESCs, iPSC wt clone, iPSC PAI-1 KO clone 3 and MEFs, as indicated. Cells were labeled with anti-Sox2 antibodies (upper panels) or anti-SSEA1 antibodies (lower panels). Same microscope fields labeled with DAPI are shown (right panels). (TIF) [file pone.0049065.s009.tif]

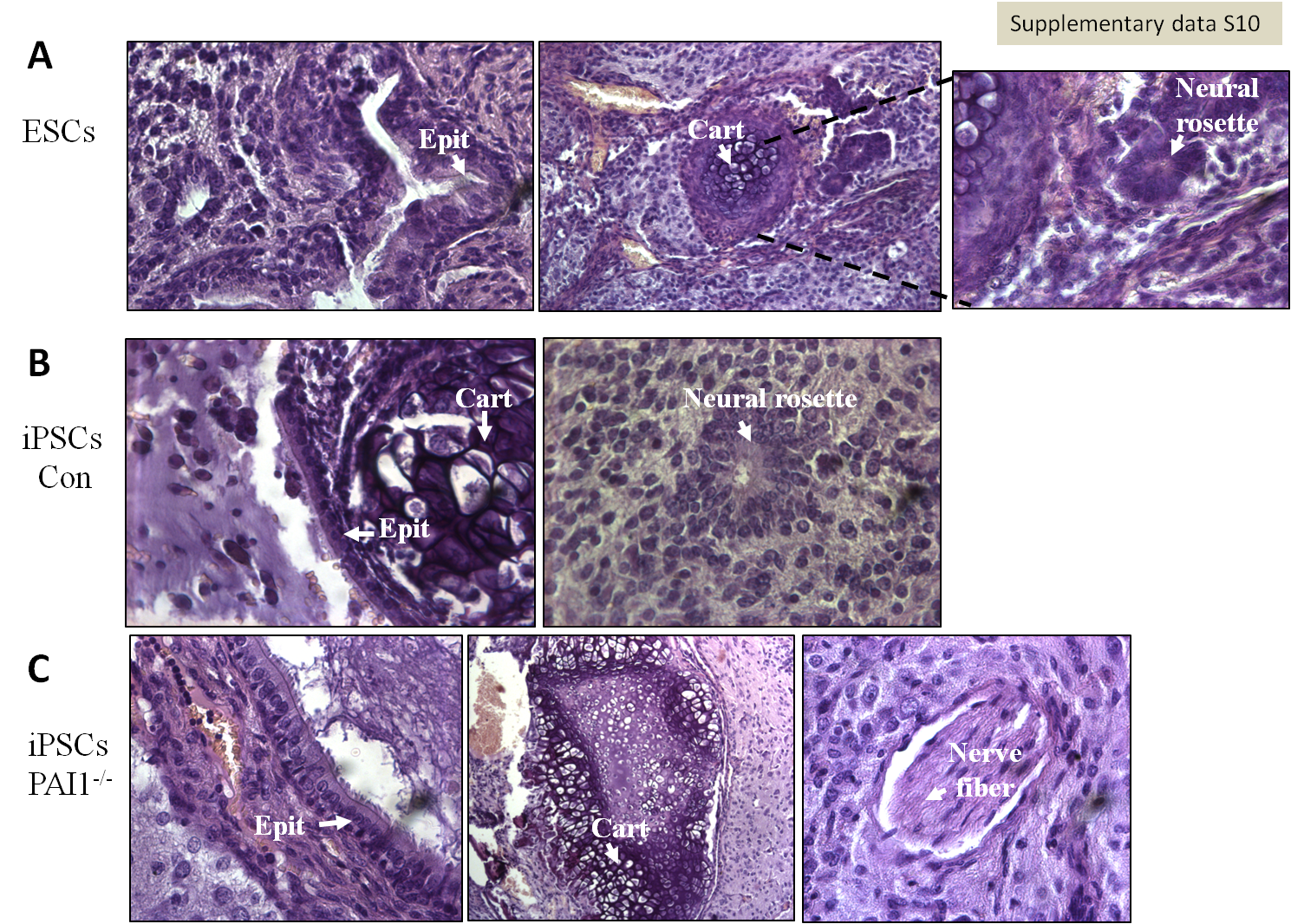

Supplement: Data S10 — histological characterization of PAI-1+/+ and −/− iPSC teratoma. CGR8 wt ESCs (A), iPSC wt clone (B), and iPSC PAI-1 KO clone 3 (C) were injected subcutaneously into nude mice. After four weeks, sections of the different teratomas were histologically analyzed. All teratomas presented differentiated tissues deriving from the three embryonic layers. Representative fields of well-differentiated tissues are shown. Arrows indicate examples of the three germ layer derivative structures: Cart = cartilage (mesoderm derivative), Epit = ciliated epithelium (endoderm derivative), and neural structures (ectoderm derivative): neural rosettes and nerve fibers. (TIF) [file pone.0049065.s010.tif]

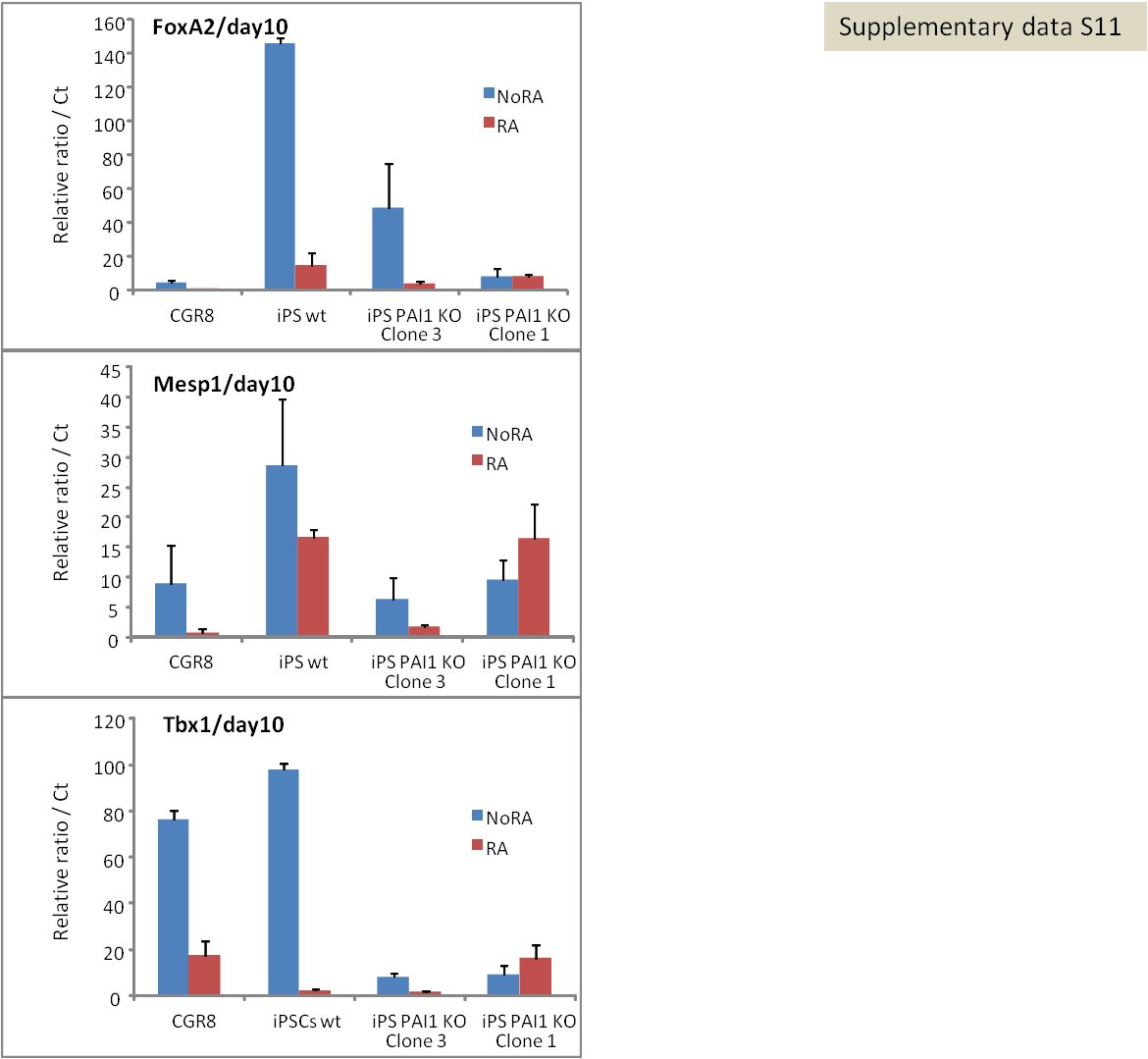

Supplement: Data S11 — characterization of PAI-1+/+ and −/− iPSCs: in vitro differentiation. Characterization of the expression of ESC commitment master genes during the differentiation process of iPSCs. Retinoic acid-treated (RA) or not (NoRA) EBs from wild type CGR8 ESCs and iPSC clones were induced to differentiate. mRNAs were extracted at day 10 and analyzed by real time RT-PCR for the expression of FoxA2, Mesp1 and Tbx1. Results are expressed in arbitrary units, with the values of wild type CGR8 at day 0 taken as 1, and are the means ± S.E.M. of at least 3 independent experiments. (TIF) [file pone.0049065.s011.tif]

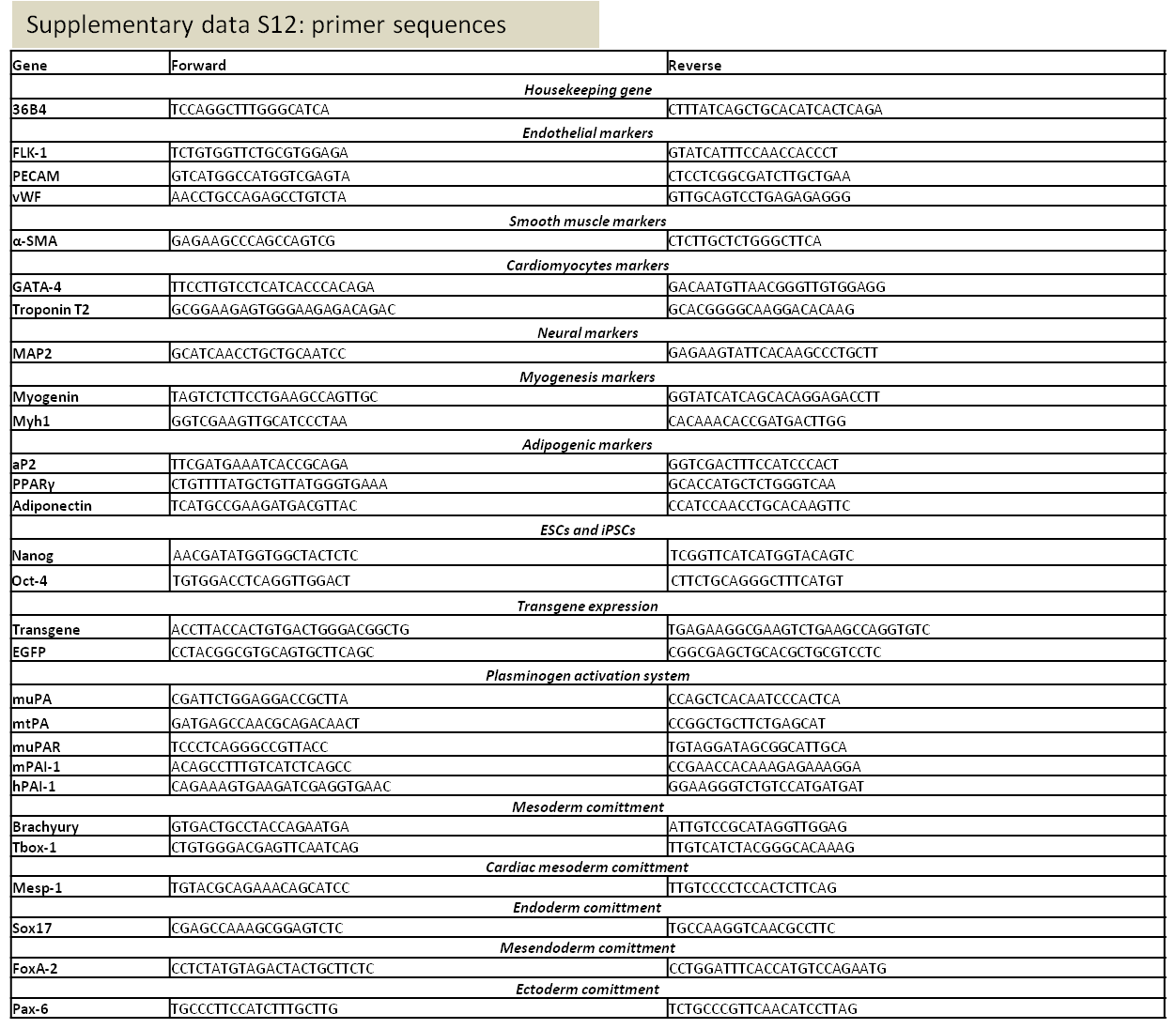

Supplement: Data S12 — Forward and reverse primer sequences of the various genes analyzed by real-time RT-PCR. (TIF) [file pone.0049065.s012.tif]
